# Verification and optimization of impact-response consistency in human thoracic targets for blunt ballistic impact testing

**DOI:** 10.3389/fbioe.2026.1929295

**Published:** 2026-09-15

**Authors:** Jiakai Wu, Song Wang, Jiaming Song, Jingpeng Wu, Bin Chen, Zhihua Cai

**Affiliations:** 1 School of Equipment Management & Support, Chinese People’s Armed Police Force Engineering University, Xi’an, China; 2 Graduate Brigade, Chinese People’s Armed Police Force Engineering University, Xi’an, China; 3 School of Mechanical and Electrical Engineering, Hunan University of Science and Technology, Xiangtan, China

**Keywords:** biomechanical response, blunt ballistic impact, finite-element model optimization, non-lethal kinetic projectile, thoracic target

## Abstract

**Introduction:**

The validation of human thorax targets for blunt ballistic impact injury assessment is a critical component in investigating their impact responses. Existing studies generally validate physical and finite-element (FE) thoracic targets independently against standardized response corridors. However, whether these two targets produce quantitatively consistent responses under identical blunt ballistic impact conditions remains insufficiently investigated.

**Methods:**

To resolve the discrepancy in impact responses between physical and digital thorax targets and establish a verification method for the consistency of blunt impact responses between physical and digital human thoracic targets for non-lethal kinetic projectiles, a 3-rib physical thoracic target with ribs made of three different materials was constructed, and the CHEST finite-element model was selected as the digital target. According to AEP-99 standards, blunt impact tests and numerical simulations of SIR-X projectiles were conducted at impact velocities of 56 m/s and 86.5 m/s. Each impact condition was tested three times, and the repeatability and uncertainty were evaluated using the coefficient of variation.

**Results:**

The coefficients of variation ranged from 2.74% to 12.20%, indicating acceptable experimental repeatability. The results show that both the physical target with PA66+GF30 ribs and the CHEST digital target meet the standard requirements for displacement-time curves and peak viscous response values. Using the physical test results as a reference, LS-OPT and the sequential response surface method were employed to optimize the Young’s modulus of costal cartilage in the digital target. After optimization, the normalized mean absolute error of the displacement response between the physical and digital targets decreased from 4.8% to 3.4% at 56 m/s and from 12.5% to 4.9% at 86.5 m/s, greatly improving consistency.

**Discussion:**

Thus, this study demonstrates that the proposed verification method effectively enhances the biomechanical reliability of digital thoracic targets. The validated physical and digital targets can provide standardized testing and technical support for thoracic injury assessment, experimental validation, and safety design of non-lethal kinetic projectiles.

## 1 Introduction

Non-lethal kinetic projectiles are weapons that use kinetic energy to strike a target and temporarily incapacitate it without causing permanent injury (Wang, 2021). Since the 1970s, they have been widely used by military and police forces to manage riots and other mass disturbances (Liu et al., 2025). As an effective supplement to conventional lethal weapons, they represent an important class of non-lethal weapons and can enhance less-lethal enforcement capability (Zhan et al., 2017). However, improper use and unclear injury mechanisms can cause severe injury or even death in scenarios of real-world impacts (Ouyang et al., 2024). The chest is one of the regions that is most often struck by non-lethal kinetic projectiles. It also contains vital organs. Severe thoracic injuries caused by blunt impacts from non-lethal kinetic projectiles have been frequently reported in actual use. In the 2008 Menglian police assault incident in Yunnan, China, police fired 18.4-mm rubber projectiles in self-defense, and the impacts to the offenders’ chests resulted in two deaths (Xiang, 2003). During the 2025 Los Angeles riots in the United States, dozens of chest injury cases caused by the police’s use of non-lethal kinetic projectiles were reported (Los Angeles Times, 2025). In the 373 injuries caused by blunt impacts from non-lethal kinetic projectiles summarized by Hubbs (Hubbs and Klinger, 2004), the chest impacts alone accounted for 19.1%, second only to abdominal impacts. These cases show that the human chest is a major region where blunt impacts from non-lethal kinetic projectiles can cause severe or even fatal injury. Therefore, chest injury caused by non-lethal kinetic projectiles is an important component of human blunt-impact injury research. It is also a key topic in the study of blunt-impact terminal effects (Wang and Wang, 2022).

To scientifically assess chest injury caused by non-lethal kinetic projectiles and improve the safety of their design and use, researchers have developed a series of human thoracic targets and conducted blunt-impact tests to simulate the biomechanical response of the human chest. For physical thoracic targets, Qi et al. (2017) used beanbag projectiles of two different calibers to impact a Hybrid III 50th-percentile male thoracic target. They recorded acceleration, displacement, and other mechanical response data to quantitatively simulate projectile-induced chest injury. Robbe et al. (2019) used different non-lethal kinetic projectiles to impact a 3-rib Ballistic Impact Dummy (3RBID) thoracic target and recorded displacement data to calculate the peak viscous response, thereby evaluating the projectile injury potential Chaufer et al. (2023a) developed the highly biofidelic SurHUByx human thoracic target and used L5 projectiles to impact it. They recorded force–time and displacement–time curves and observed rib fractures to simulate the severity of human chest injury. For digital thoracic targets, Thota et al. (2014) developed a simplified human thorax finite-element model, MTHOTA, for blunt injury assessment. Wang et al. (2020) improved this model, developed and validated the MTHOTA-I finite-element model, and used it to simulate rubber projectile blunt impacts to the chest and assess the injury risk. Bracq et al. (2019) developed the HUByx human thorax finite-element model and conducted simulations of combined projectile impacts to evaluate the thoracic organ injury. Based on the Hybrid III finite-element model, Kebbab et al. (2026) proposed a simplified thorax model composed of muscle, bone, and lung tissue, named STH-FEM. They analyzed its mechanical response under different impact velocities through numerical simulation and validated it according to AEP-99. The results showed that the simplified model met the standard requirements and was suitable for basic injury assessment and ballistic safety design. Roberts et al. (2007) developed a fifth-percentile male human torso finite-element model, named the Human Torso Finite-Element Model (HTFEM). They performed simulations under dynamic conditions such as high-speed impact and compared the results with animal and human cadaver test data to assess human injury severity. Previous studies have established various physical and finite-element thoracic targets for evaluating blunt impact responses caused by non-lethal kinetic projectiles. Physical targets, such as 3RBID and other biofidelic targets, provide standardized experimental measurements and have been widely validated using the displacement corridor and viscous response criteria specified in AEP-99. However, these models mainly focus on experimental qualification and provide limited information on internal biomechanical mechanisms. In contrast, finite-element thoracic models, including simplified and anatomically detailed models, can describe tissue-level responses and injury mechanisms. Nevertheless, the material parameters of these models are commonly derived from literature data or empirical assumptions, and their transient impact responses are not always calibrated against corresponding physical tests under identical loading conditions. Therefore, existing studies have primarily addressed the validation of individual physical or numerical targets, while the quantitative consistency between physical and finite-element thoracic targets remains insufficiently investigated.

Previous studies have improved thoracic finite-element models through material property adjustment, component-level validation, sensitivity analysis, and experiment-based parameter calibration. Zeng et al. (2021) adopted a hierarchical validation strategy to improve the musculoskeletal response of a human thorax model at the rib, rib cage, and whole thorax levels. Chaufer et al. (2023b) modified and homogenized the mechanical properties of the thoracic bones and costal cartilage while preserving their equivalent bending stiffness and evaluated the resulting thoracic deflection under blunt impact loading. Other studies (Wang et al., 2016; Iraeus et al., 2020) have investigated the sensitivity of thoracic impact responses to the material properties of the skeletal and soft-tissue components or have used LS-OPT to calibrate the selected thoracic tissue parameters against experimental responses. However, the objectives, loading conditions, model structures, experimental targets, and optimized parameters differed substantially among these studies.

At the standardization level, the North Atlantic Treaty Organization (NATO) issued AEP-99, a standardized document for assessing thoracic injury caused by blunt impacts from non-lethal kinetic projectiles, in 2017 and revised it in 2021 (NATO Standardization Office NSO, 2017; NATO Standardization Office NSO, 2021). This document uses both the displacement–time corridor and the peak viscous criterion VC_max_ after projectile impact as validation criteria for thoracic targets. It also provides two validation methods based on blunt impacts from L5 and SIR-X projectiles. When a thoracic target is impacted by a test projectile at the specified velocity, it passes validation if its chest-wall displacement–time curve falls within the AEP-99 corridor and its VC_max_ value lies within the specified range. This validation method is applicable to both physical and digital thoracic targets. At present, the criteria specified in AEP-99 have been widely used for thoracic target validation. Based on this standard, Robbe et al. (2023) used composite non-lethal kinetic projectiles to impact 3RBID and BTTR physical thoracic targets. They found that the 3RBID target met the validation requirements, whereas the impact response of the BTTR target was slightly higher than the specified corridor and data range. Zhang et al. (2025a) conducted L5 projectile blunt-impact simulations on three thorax finite-element models, namely, Hybrid III, THUMS, and CHEST. These simulations were used to verify whether the models satisfied the biomechanical response corridor of the human thorax, thereby supporting the selection of thoracic targets for non-lethal kinetic projectile blunt-impact testing.

Overall, existing studies have established a research basis that combines physical thoracic target testing, digital thoracic target simulation, and standardized validation for chest injury assessment of non-lethal kinetic projectiles. This provides important support for characterizing the biomechanical response of the human chest under blunt impact. Although previous studies have established physical and finite-element thoracic targets for blunt-impact assessment, these targets have generally been developed and validated independently. Most studies have focused on determining whether an individual target satisfies a prescribed displacement corridor or viscous response range. However, compliance with the same validation criteria does not necessarily indicate that physical and finite-element targets produce quantitatively consistent transient responses under identical impact conditions. Moreover, the material parameters of finite-element thoracic models are commonly obtained from literature data or empirical assumptions, with limited calibration against corresponding physical experiments. Therefore, a scientific gap remains in establishing a systematic method that first verifies both targets under standardized conditions and then quantitatively evaluates and improves their impact response consistency through experiment-informed parameter optimization.

To address this scientific gap, the present study develops a framework for verifying and improving the consistency of impact response between physical and finite-element thoracic targets, Specifically, the physical thoracic target and the CHEST finite-element model are first validated under the same AEP-99 impact conditions, their transient responses are then quantitatively compared, and a sensitive material parameter of the finite-element model is finally optimized using the physical test response as the calibration reference. Moreover, this study proposes a method for verifying impact response consistency between physical and digital thoracic targets under blunt impacts from consistency-verified physical and digital SIR-X projectiles, aims to construct and screen a 3-rib physical thoracic target according to AEP-99, validate the CHEST FE model under the same projectile and velocity conditions, quantify the physical–digital response discrepancy, and identify and optimize a sensitive FE material parameter to improve displacement and VC consistency. The specific steps are as follows. First, the physical and digital thoracic targets are validated under the impact conditions specified in AEP-99. Then, key biomechanical parameters of the digital thoracic target are optimized using the impact response curve of the physical target as the reference so that the digital target meets the consistency requirement. Finally, from the perspective of practical application, a safety design process for new non-lethal kinetic projectiles is proposed based on impact response consistent thoracic targets. For the physical target, the new 3-rib thorax model designed by Luo et al. (2026) is used. Based on its original structure and material system, the model is reconstructed using simulated ribs with three different mechanical properties and manufacturing processes, together with a simulated skin–fat–muscle structural layer. The three physical models with different rib materials are then validated under the AEP-99 impact conditions, and the 3-rib thorax model that meets the standard requirements is selected. For the digital target, the anatomically detailed CHEST human thorax finite-element model developed by Cai et al. (2023) is used and validated under the specified impact conditions. The CHEST model was selected because it was developed based on high-resolution CT data of a 50^th^-percentile Chinese adult man and includes detailed representations of the ribs, sternum, thoracic vertebrae, costal cartilage, ligaments, skin, muscles, heart, and lungs. Compared with dummy-based or simplified thoracic models, the CHEST model can provide both the global chest wall responses required for AEP-99 validation and local tissue-level mechanical responses relevant to thoracic injury assessment. In addition, our previous study demonstrated that the CHEST model satisfied the AEP-99 response requirements under blunt impact conditions. Therefore, this model provides an appropriate combination of anatomical biofidelity, prior validation, and tissue-level response capability for evaluating and optimizing the consistency between physical and finite-element thoracic targets. Its selection was based on the objectives of the present study and does not imply that it is universally superior to all other validated thoracic models. The impact responses of the validated physical and digital thoracic targets are then compared. Based on the impact response curve of the physical target, LS-OPT is used to optimize the digital target parameters so that the physical and digital thoracic targets meet the requirement for impact response consistency. This study improves the testing accuracy and reliability of the digital thoracic target through impact response consistency verification. It also supports test qualification and promotes the digital twinning of thoracic targets for blunt impact testing, thereby assisting in the development of the optimized design of new projectiles. In addition, it provides a technical route and methodological reference for other physical and digital targets used in non-lethal kinetic projectile testing, such as head targets and multilayer skin targets.

## 2 Materials and methods

### 2.1 Construction of the physical thoracic target and test projectiles

In AEP-99, the 3RBID is defined as the standardized physical thoracic target for non-lethal kinetic projectile testing. This physical target technology originates from the thoracic surrogate structure of the Biofidelic Side Impact Dummy (BioSID), which was developed by Lyons et al. (Grewal et al., 1994) to assess human injury in vehicle side impacts. Based on BioSID, Bir (2000) extracted a 3-rib structural unit and combined it with the appropriate loading surface of the Hybrid III dummy. He developed a 3-rib Chest Structure (3-RCS) consisting of three ribs, a spine box, damping material, support components, and displacement sensing components. This structure was used to measure dynamic chest deformation under local high-speed impact and to evaluate chest injury caused by non-lethal kinetic projectiles. Robbe et al. (2014) further developed the 3-RCS into the 3RBID and applied it to thoracic impact studies of non-lethal kinetic projectiles. The 3RBID was later adopted by AEP-99 as the standardized physical thoracic target. Its development process is shown in Figure 1.

**FIGURE 1:**
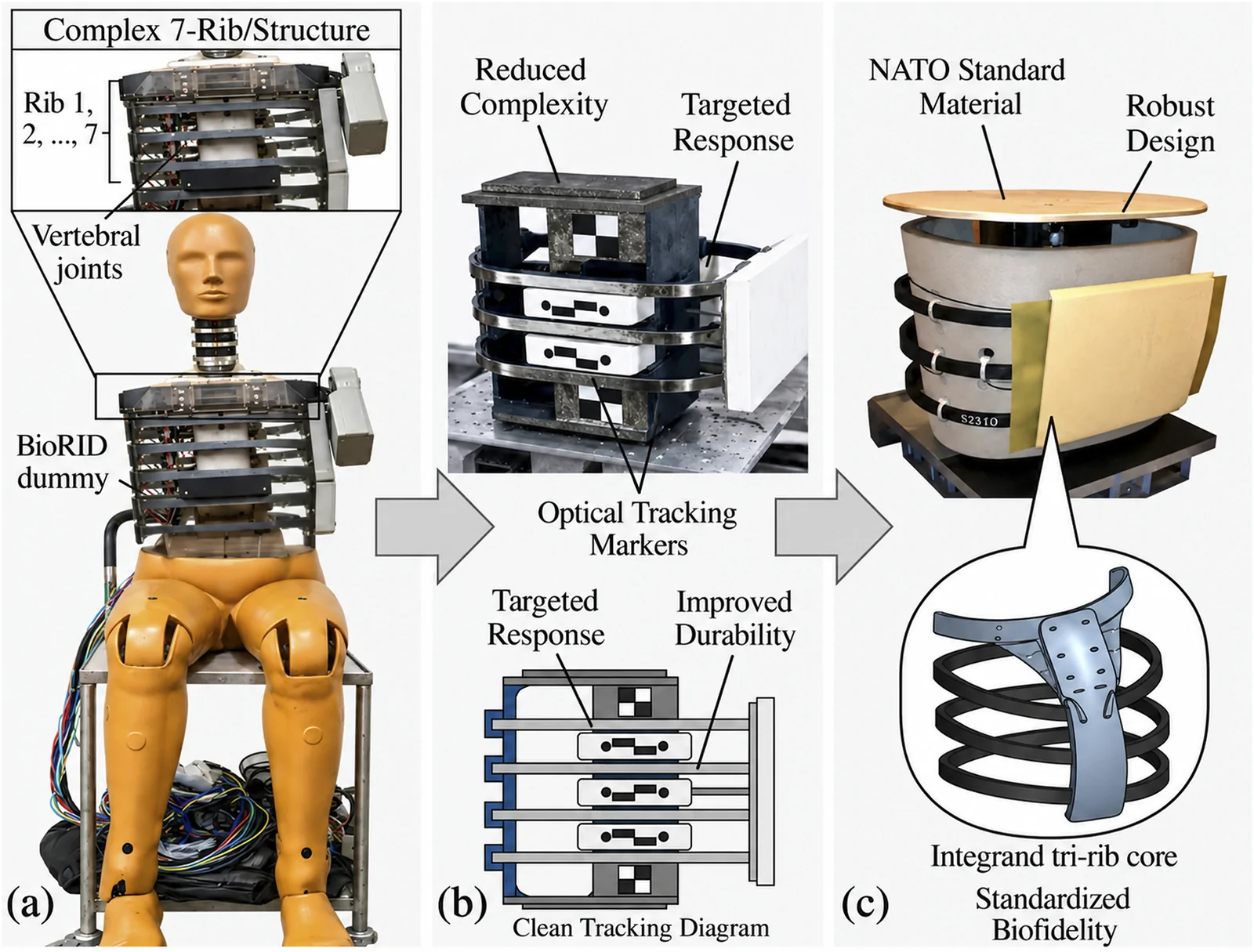
Development history of 3-rib Ballistic Impact Dummy: **(a)** Biofidelic Side Impact Dummy; **(b)** 3-rib Chest Structure; **(c)** 3-rib Ballistic Impact Dummy.

Luo et al. (2026) developed an integrated 3-rib physical thorax model based on the 3RBID. The model has an adjustable structure and is compatible with simulation validation. It showed a good impact response under AEP-99 conditions. Compared with physical models such as Skeletal and Human Tissue Impact Model (SHTIM), its response was closer to the centerline of the standard corridor, supporting the engineering application of standardized physical thoracic targets.

The geometric model of the physical thoracic target used in this study was based on the 3-rib thorax model developed by Luo et al. (2026). The model consists of a skin layer, a soft-tissue layer, and a rib-bearing layer. Its main components include an aluminum alloy bracket, engineering plastic-simulated ribs and spine box, polyurethane-simulated soft tissue, and silicone-simulated skin. The structural composition and key dimensions of the model are shown in Figure 2.

**FIGURE 2:**
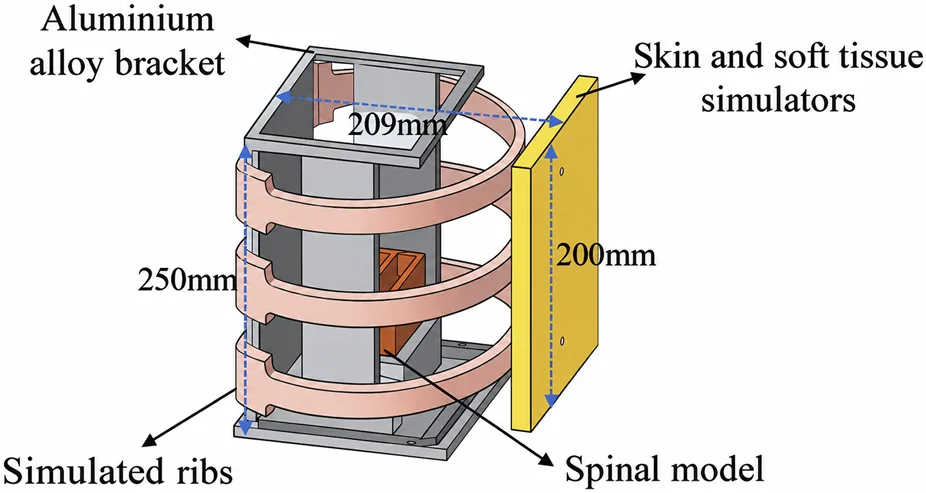
Structural composition and key dimensions of the 3-rib thorax model.

Based on the experimental requirements of this study, the impact mechanical characteristics of the geometric model, and its repeatable assembly feature, the original geometry and dimensions were retained. The design also followed the typical anatomical parameters of the fourth to sixth human ribs in the original structure. Two modifications were made during target construction. First, according to the basic data in Chinese Adult Human Body Dimensions (GB/T 10000—2023) (Zhang et al., 2023), the chest wall was treated as a multilayer structure composed of skin, fat, and muscle. The original skin–soft tissue, or subcutaneous tissue, layer was, therefore, refined into a skin–fat–muscle layer. Because the chest-wall thickness differs significantly among intercostal regions, an equivalent simplified modeling method was used. The simulated skin layer was set to 2.0 mm, which falls within the average Chinese skin thickness of 2.31 ± 1.06 mm. The simulated fat layer was set to 0.8 mm to represent the equivalent layered behavior of superficial chest-wall fat under impact loading. The simulated muscle layer was set to 1.2 mm to represent the equivalent thickness of the superficial load-bearing muscle tissue in the intercostal region. This refined layered structure helps observe specific soft-tissue damage more clearly. Second, the manufacturing process and material parameters of the simulated ribs were changed. Three types of simulated ribs were used to assemble targets with different material properties; PA66+GF15, PA66+GF30, and GFPA12GF were selected as candidate rib materials based on their biomechanical relevance, manufacturability, and differences in structural stiffness. The nominal Young’s moduli values range from 12.9 to 19.3 GPa, which is close to the reported modulus range of adult human rib cortical bone. In particular, the Young’s moduli of PA66+GF15 and PA66+GF30 are within commonly reported ranges, while GFPA12GF provides relatively low stiffness for evaluating the influence of rib stiffness on the thoracic impact response. Glass fiber-reinforced polyamides also provide relatively high specific stiffness and are suitable for repeatable component fabrication. PA66+GF15 and PA66+GF30 can be manufactured by machining, whereas GFPA12GF can be produced by additive manufacturing. The three materials, therefore, serve as candidate ribs with different stiffness levels and manufacturing methods. Their suitability was subsequently evaluated using the displacement response and viscous response criteria specified in AEP-99. The adjusted materials and material parameters of the physical thorax model, based on Luo et al. (2026) and relevant human thoracic biomechanical parameters, are listed in Table 1.

**TABLE 1:** Adjusted materials and material parameters of the physical thoracic model.

Component	Material	Manufacturing process	Thickness/mm	Density(ρ)/kg·m−3	Poisson’s ratio (*ν*)	Young’s modulus (E)/GPa
Simulated skin layer	Silicone	Calendering and vulcanization	2.0	1,100	0.48	0.001
Simulated fat layer	Non-porous foam	Closed-cell foaming	0.8	100	0.32	0.03
Simulated muscle layer	EVA foam	Cross-linked foaming	1.2	150	0.42	0.2
Simulated rib 1	PA66+GF15	Lathe machining	6.0	1,650	0.35	15.0
Simulated rib 2	PA66+GF30	Lathe machining	6.0	1,290	0.37	19.3
Simulated rib 3	GFPA12GF	3D printing	6.0	1,200	0.31	12.9
Bracket	Aluminum alloy	Lathe machining	—	2,700	0.33	69.0

To assess the biomechanical relevance of the selected surrogate rib materials, their nominal mechanical properties were compared with published experimental data for adult human rib cortical bone, as summarized in Table 2. Dynamic tensile coupon testing of human rib cortical bone reported an average Young’s modulus of 13.9 GPa, a yield stress of 93.9 MPa, an ultimate stress of 124.2 MPa, and an ultimate strain of 2.7% (Kemper et al., 2005). Another dynamic tensile coupon testing reported an average Young’s modulus of 13.5 GPa under quasistatic loading, with failure stresses of 112.0 MPa under quasistatic loading and 124.6 MPa under dynamic loading (Katzenberger et al., 2020). The Young’s moduli of the three candidate rib materials ranged from 12.9 to 19.3 GPa and were, therefore, comparable to the values reported for adult human rib cortical bone.

**TABLE 2:** Comparison of the mechanical properties of the surrogate rib materials and adult human rib cortical bone.

Material or specimen	Density (kg/m^3^)	Poisson’s ratio	Young’s modulus (GPa)	Yield stress (MPa)	Ultimate or failure stress (MPa)	Ultimate or failure strain (%)	Data source
PA66+GF15	1,650	0.35	15.0	NR	NR	NR	Present study
PA66+GF30	1,290	0.37	19.3	NR	NR	NR	Present study
GFPA12GF	1,200	0.31	12.9	NR	NR	NR	Present study
Human rib cortical bone and dynamic tension	NR	NR	13.9	93.9	124.2	2.7	Kemper et al. (2005)
Human rib cortical bone and quasistatic tension	NR	NR	13.5	NR	112.0	NR	Katzenberger et al. (2020)
Human rib cortical bone and dynamic tension	NR	NR	NR	NR	124.6	NR	Katzenberger et al. (2020)

NR indicates that the corresponding property was not reported in the cited study or was not available for the present surrogate material. The properties of human rib cortical bone vary with donor age, anatomical region, loading mode, and strain rate. Therefore, the comparison primarily reflects the similarity in nominal elastic stiffness rather than complete material equivalence.

However, similarity in Young’s modulus does not imply complete constitutive equivalence. Human rib cortical bone is heterogeneous and anisotropic, and its mechanical properties vary with age, anatomical location, loading mode, and strain rate. In contrast, the candidate materials are glass fiber-reinforced polyamides whose responses may also be affected by fiber orientation and manufacturing processes. Therefore, the comparison was used only as an initial stiffness-based screening criterion. The final selection of the surrogate rib material was determined from the displacement response and viscous response criteria specified in AEP-99.

Strain rate dependency is relevant to the present impact conditions because the loading process occurs within milliseconds. Previous studies have shown that the stiffness, strength, and failure behavior of human rib cortical bone may vary with the loading rate. Glass fiber-reinforced polyamides may also exhibit rate dependent mechanical behavior because of their polymer matrix and fiber-reinforced structure. However, dynamic constitutive data at strain rates representative of the present blunt impact conditions were not available for all three candidate rib materials. Therefore, the nominal Young’s moduli listed in Table 2 were used as first-order indicators of structural stiffness rather than as complete descriptions of their dynamic constitutive behavior. The suitability of each candidate material was subsequently evaluated using the displacement and viscous responses measured under the AEP-99 impact conditions.

After the aluminum alloy bracket, laser displacement sensor, simulated ribs, simulated skin, fat, and muscle layers were fabricated, the thorax model components were assembled with bolts based on the geometric structure shown in Figure 1. A laser displacement sensor was fixed to the support bracket behind the central rib to measure its displacement. The constructed physical thorax model and its components are shown in Figure 3.

**FIGURE 3:**
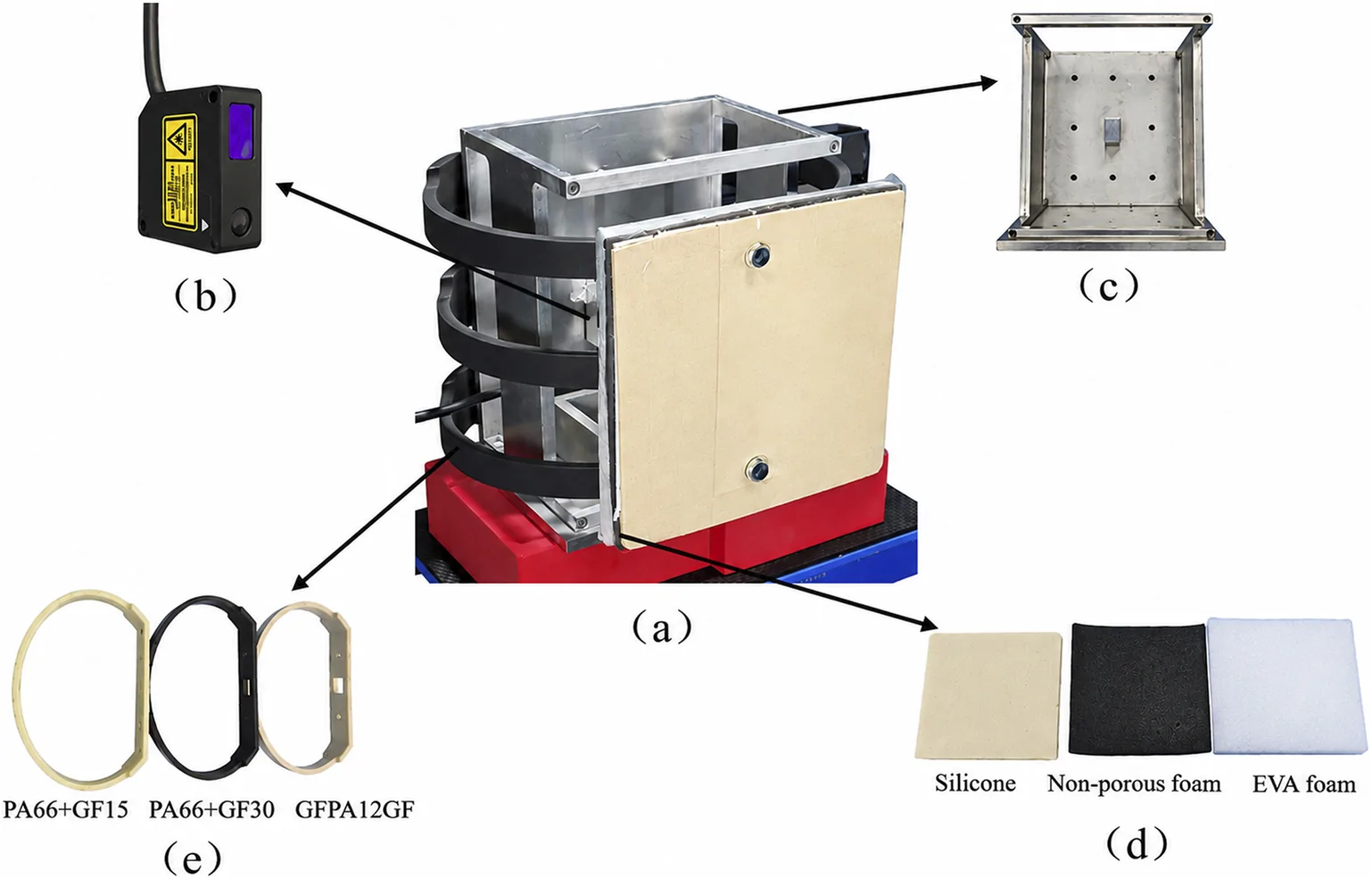
3-rib thorax model and its components. **(a)** 3-rib thorax model; **(b)** LTM5 laser displacement sensor; **(c)** aluminium; **(d)** simulations of skin, fat, and muscle layers; **(e)** simulated ribs.

The test projectile was the SIR-X composite non-lethal kinetic projectile specified in AEP-99. It had a mass of 32 g and a caliber of 40.2 mm. The projectile consisted of a polyurethane foam nose and an engineering plastic body. The foam nose was fabricated by foaming using polyurethane foam with a hardness of 60 HOO. The projectile body was made of PVDF by lathe machining because of its high impact resistance and good mechanical performance. The geometry and physical prototype of the projectile are shown in Figure 4.

**FIGURE 4:**
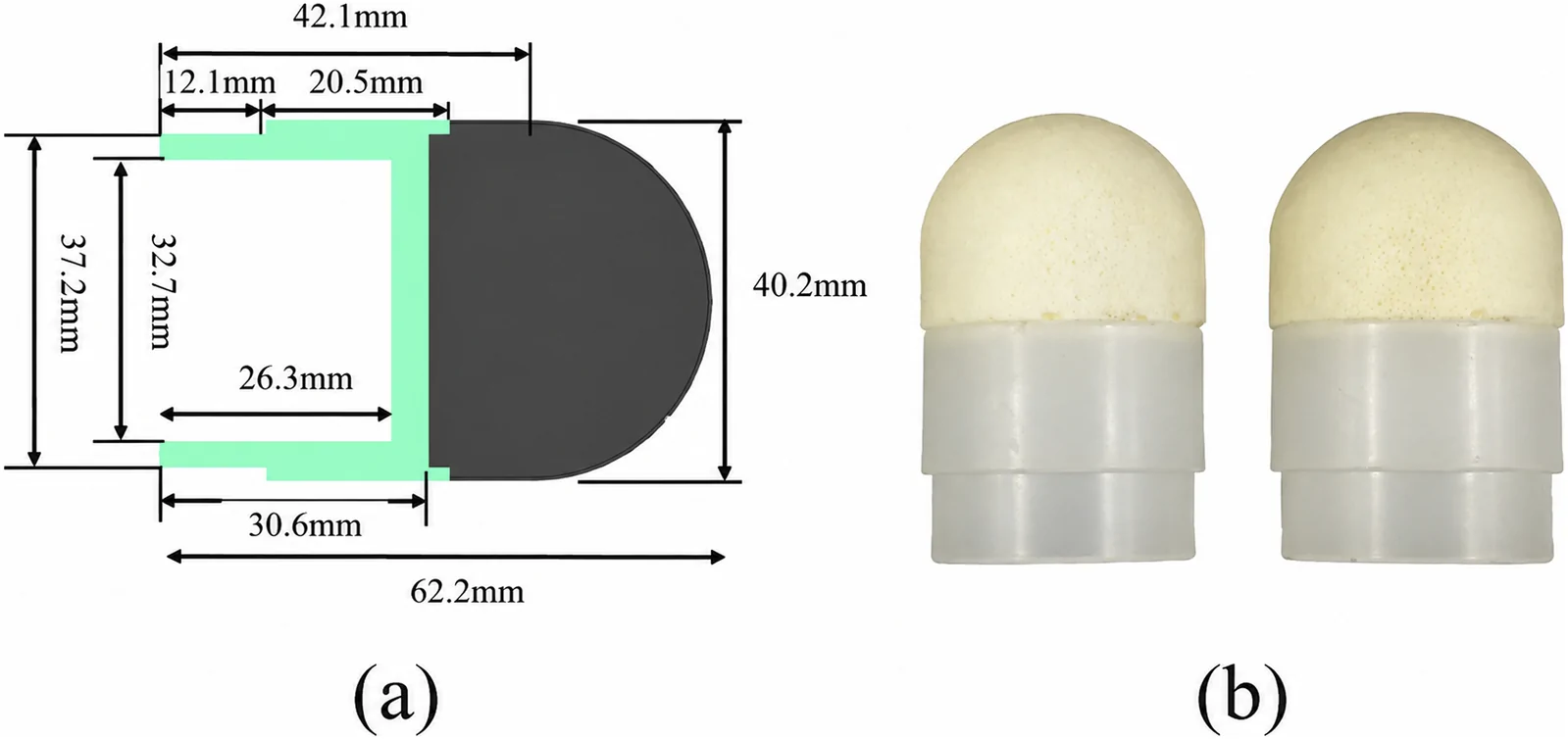
Geometric dimensions and physical item of the SIR-X projectile. **(a)** Geometric dimensions; **(b)** physical item.

### 2.2 Blunt ballistic impact experimental setup

After the physical thoracic target and standardized test projectile were constructed, blunt-impact tests were conducted on the physical thoracic target using the blunt impact platform. A laser displacement sensor was used to record chest-wall displacement after the impact, and a high-speed camera was used to capture the dynamic response of the thoracic target. Tests were performed under the two impact velocities specified in the standard. The simulated rib material was then changed, and repeated tests were carried out to obtain the displacement–time curves of the thoracic targets with three different simulated rib materials. The corresponding VC–time curves were then calculated. The detailed calculation methods are given in Equations 7, 8 of the *Results and Discussion* section. These results were compared with the response corridors and VC_max_ ranges specified in AEP-99 to select the thoracic target that met the standard requirements.

The nominal impact velocities of 56 ± 2 m/s and 86.5 ± 2.5 m/s were selected because they are the prescribed validation conditions for the SIR-X projectile in AEP-99. These two conditions represent different levels of impact loading and are used to determine whether a thoracic target satisfies the specified displacement and viscous response requirements. This study performs a standard-based validation and evaluates the response consistency between the physical and finite-element thoracic targets under identical conditions. Therefore, additional velocities outside the AEP-99 validation conditions were not included.

The experiment was conducted on a blunt impact platform. The main devices included a ballistic air cannon, a velocity measurement system (Light curtain target), a closed target chamber, a physical thoracic target, a high-speed camera, fill lights, and an integrated operating platform. Before the test, the physical thoracic target was placed in the closed target chamber. A light-screen chronograph was installed at the muzzle of the air cannon to accurately record the initial projectile velocity. The laser displacement sensor was fixed to the target support behind the central rib and connected to the data acquisition system through a signal cable. It was used to record the chest-wall displacement signal and obtain the displacement–time curve of the thoracic target. A high-speed camera was placed beside the target chamber to capture the dynamic response of the thoracic target after the projectile’s impact. Because the short exposure time required for high-speed imaging reduces image brightness, fill lights were used to improve the illumination. The camera focus was then adjusted to ensure clear images are obtained. Finally, the projectile was loaded into the air cannon for testing. The schematic of the physical thoracic target impact test is shown in Figure 5, and the blunt ballistic impact platform and its components are shown in Figure 6.

**FIGURE 5:**
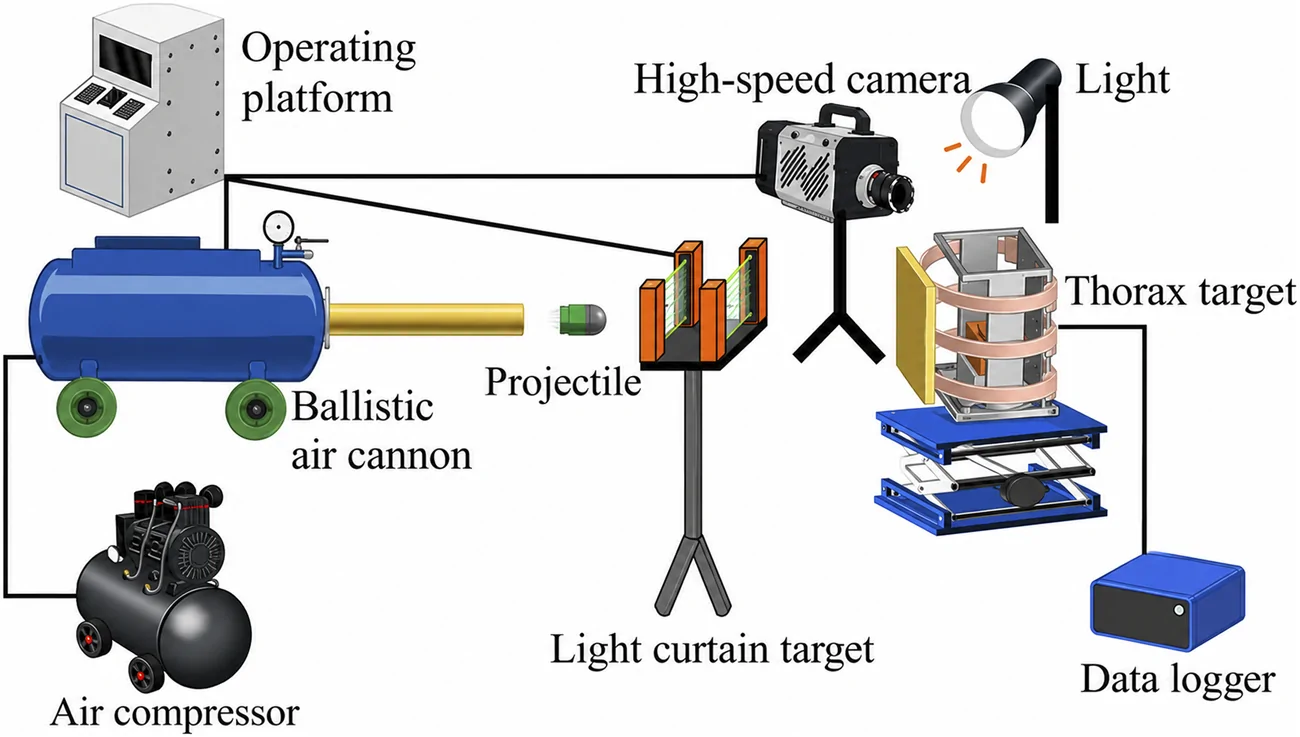
The schematic of the physical thoracic target impact test.

**FIGURE 6:**
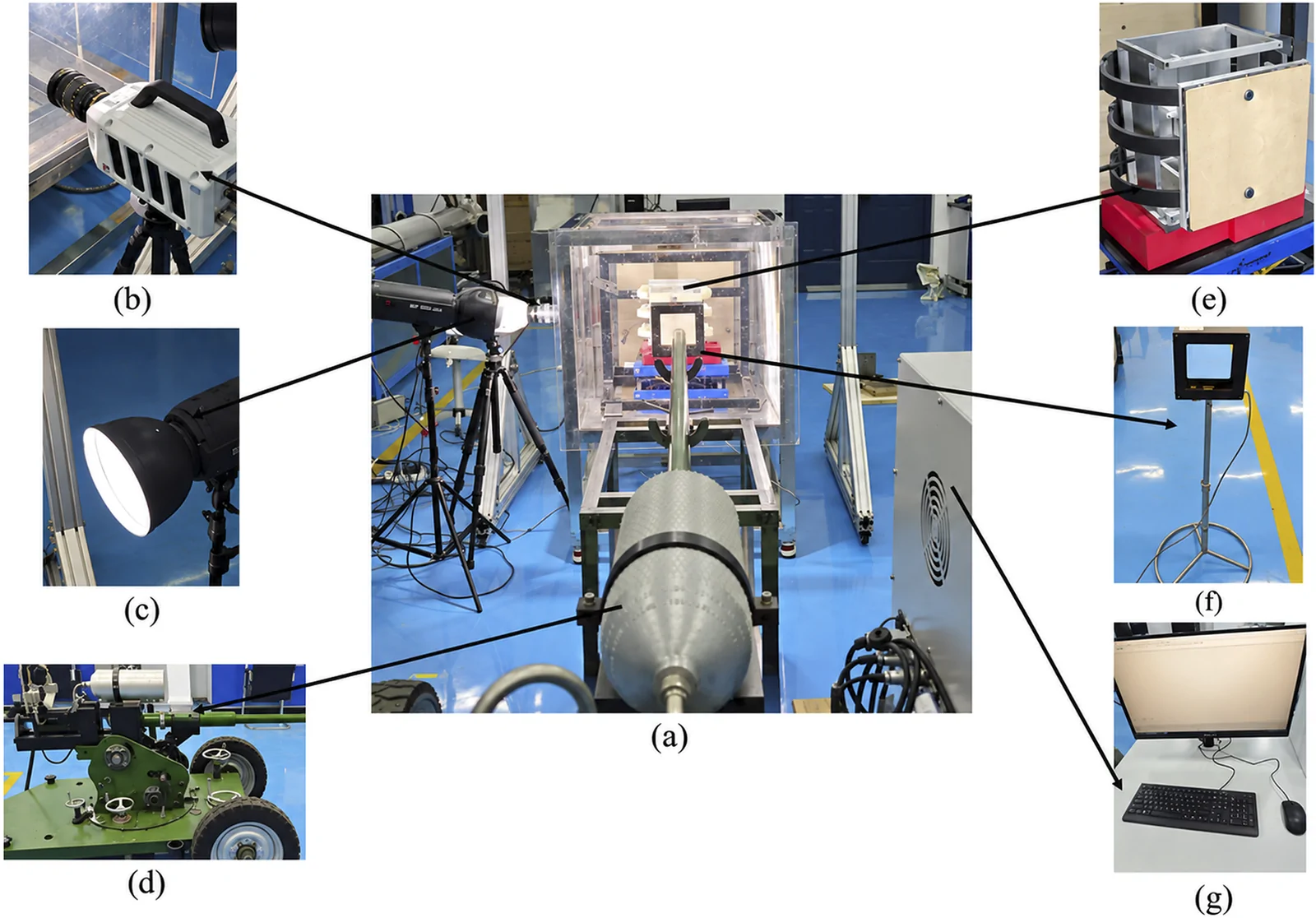
Blunt impact platform and its components. **(a)** Blunt ballistic impact platform. **(b)** High-speed camera. **(c)** Light. **(d)** Ballistic air cannon. **(e)** Thorax target. **(f)** Light curtain target. **(g)** Operating platform.

The principal measurement instruments and their specifications are summarized in Table 3. The displacement of the central rib was measured using an LTM5-085WEA laser displacement sensor manufactured by Hongchuan Technology. The sensor was mounted behind the central rib and aligned with the impact direction. Its stated measurement accuracy was 0.02 mm, and the displacement signal was acquired at a sampling frequency of 50 kHz. The measured displacement was used to obtain the displacement time response and to calculate the viscous response.

**TABLE 3:** Specifications and functions of the principal measurement instruments.

Instrument	Manufacturer and model	Measured quantity	Accuracy or resolution	Sampling or recording rate	Function in the experiment
Laser displacement sensor	Hongchuan Technology, LTM5-085WEA	Central rib displacement	Measurement accuracy: 0.02 mm	50 kHz	Quantitative measurement of the chest wall displacement response and calculation of the viscous response
High-speed camera	Revealer, S1310 M/C	Impact process and target deformation images	Not used for quantitative displacement measurement	10,000 frames/s	Qualitative observation of projectile contact, thoracic compression, rebound, recovery, and visible rib damage
Light-screen chronograph	Weike Technology,wks-2T	Projectile velocity	Velocity accuracy: ±0.2%; timing resolution:1 μs	—	Measurement of projectile exit velocity immediately before the impact

The velocity ranges of 56 ± 2 m/s and 86.5 ± 2.5 m/s represent the prescribed experimental velocity windows rather than the measurement accuracy of the light-screen chronograph. The high-speed camera was used only for qualitative observation, while quantitative displacement data were obtained from the laser displacement sensor.

A Revealer high-speed camera was operated at 10,000 frames per second to record the projectile impact process and the dynamic deformation of the thoracic target. The high-speed camera images were used to identify the compression, rebound, and recovery stages and observe visible structural damage. The camera was not used for quantitative displacement measurement. Therefore, camera calibration and spatial image resolution did not contribute directly to displacement or viscous response calculations.

The projectile velocity was measured using a light-screen chronograph positioned near the muzzle of the ballistic air cannon. The manufacturer-specified velocity accuracy was ±0.2%, and the timing resolution was 1 μs. Before the formal experiments, three calibration shots for each nominal velocity condition were performed to verify that the measured velocities remained within the prescribed ranges. Because the distance between the velocity measurement position and the thoracic target was approximately 30 mm, the measured exit velocity was taken as the impact velocity.

Measurement uncertainty was estimated from the stated accuracy of the laser displacement sensor. The sensor accuracy was ±0.02 mm. Assuming a rectangular distribution, the corresponding standard uncertainty was 0.0115 mm. Because the measured displacement was normalized using a chest depth scaling factor of 236/209, the propagated standard uncertainty of the normalized displacement was 0.0130 mm. Using a coverage factor of *k* = 2, the expanded uncertainty of the normalized displacement was approximately ±0.026 mm.

The high-speed camera was used only for qualitative observation of the impact process and was, therefore, was not used in the quantitative uncertainty analysis. The prescribed velocity ranges of 56 ± 2 m/s and 86.5 ± 2.5 m/s represent the experimental acceptance ranges rather than the measurement accuracy of the light-screen chronograph.

According to the AEP-99 validation requirements for thoracic targets, the test projectile impacted the central rib of the thoracic target at velocities of 56 ± 2 m/s and 86.5 ± 2.5 m/s. Before the formal tests, three calibration tests were conducted at each velocity condition to ensure that the projectile’s impact velocity met a confidence level of at least 90%. The calibration results showed that air cannon pressures of 1.24 ± 0.03 Mpa and 2.05 ± 0.05 Mpa corresponded to impact velocities of 56 m/s±2 m/s and 86.5 m/s±2.5 m/s, respectively. Therefore, during the tests, the air cannon pressure was set to 1.24 MPa and 2.05 MPa through the integrated operating platform. The projectile was fired after the pressure reached the preset value. It passed through the light curtain target, struck the central rib of the thoracic target, and then fell into the target chamber. The displacement sensor recorded the displacement signal after impact. The light curtain target recorded the projectile exit velocity. Because the vertical distance from the muzzle to the thoracic target was only 30 mm, the exit velocity was taken as the impact velocity. The high-speed camera captured the instantaneous motion of the projectile during impact with the physical thoracic target. All recorded data were transmitted to the integrated operating platform through data cables for storage and subsequent processing.

To reduce the experimental error, each impact condition was tested three times. The impact velocity, peak normalized displacement, and maximum viscous response were calculated separately for each test. The results are presented as mean and standard deviation. The coefficient of variation was also calculated to evaluate the experimental repeatability. The formula for calculating the coefficient of variation is shown in Equation 1.

$$CV=\frac{s}{\bar{x}}\times 100\%$$ (1)

where $\bar{x}$ is the mean value and s is the sample standard deviation. Because only three repeated tests were conducted for each condition, descriptive statistics were used rather than formal hypothesis testing.

### 2.3 Construction of the digital thoracic target and test projectiles

The digital thoracic target for non-lethal kinetic projectile testing is a human thorax finite-element model used in impact testing. In this study, the anatomically detailed CHEST human thorax finite-element model developed by Cai et al. (2023) was used as the digital thoracic target. This model was built based on the thoracic dimensions of a 50th-percentile Chinese adult man. High-resolution CT was first used to obtain thoracic DICOM images. The three-dimensional geometry was then segmented and reconstructed in Mimics, and the surface was optimized in Geomagic. The mesh was generated in HyperMesh, and material properties and boundary conditions were assigned to obtain the final human thorax finite-element model. The model includes the sternum, ribs, vertebrae, heart, lungs, skin, muscles, and other tissues. The abdominal tissues are represented by muscle. It contains 3,634 shell elements, 135,503 solid elements, and 120,084 nodes. The muscle, heart, and lung tissues are modeled as viscoelastic materials, while the other organs are modeled as elastic materials. Its internal structure is shown in Figure 7.

**FIGURE 7:**
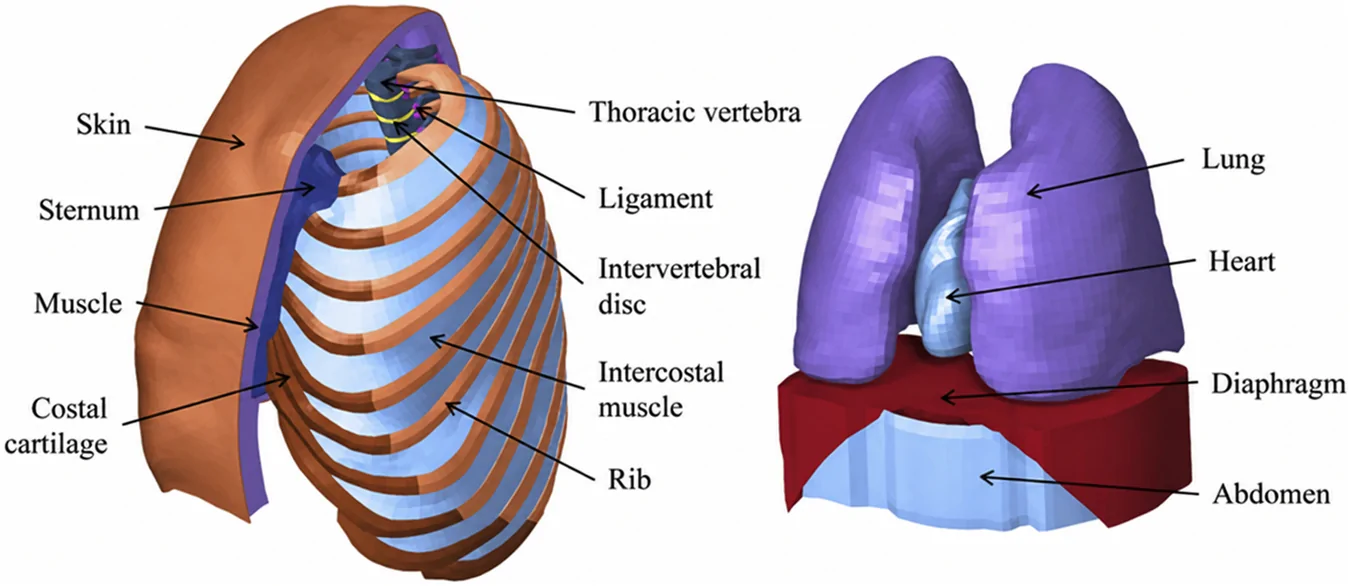
Schematic diagram of the digital thorax model.

The CHEST thorax finite-element model was first used to assess human thoracic injury caused by blast waves and high-speed fragments (Cai et al., 2023). It was later applied to research on blunt thoracic injury. In previous work, our research group conducted simulated impact tests on the Hybrid III, THUMS, and CHEST thorax finite-element models using a digital PVC projectile according to AEP-99 requirements (Zhang et al., 2025a). The results showed that the Hybrid III and CHEST thorax finite-element models met the impact condition requirements specified for the PVC digital projectile and could be used to assess human thoracic injury caused by blunt impacts from non-lethal kinetic projectiles. The CHEST finite-element model also showed higher biofidelity in anatomical structure and material parameters. Xiao et al. (2025) also used the CHEST finite-element model to evaluate rib and lung injuries caused by impacts of digital composite non-lethal kinetic projectiles. They also fitted the probability of AIS 2–3 injury under different projectile impact conditions. These findings indicate that, compared with the 3RBID finite-element model, the CHEST finite-element model has more complete internal organs and an anatomical structure that better matches the characteristics observed among Chinese humans. It can, therefore, support the qualitative analysis of overall thoracic injury and quantitative assessment of local visceral tissue injury.

The digital test projectile used the SIR-X digital projectile specified in AEP-99. Its geometry and structure were identical to those of the physical projectile. The structure of the SIR-X digital projectile finite-element model is shown in Figure 8.

**FIGURE 8:**
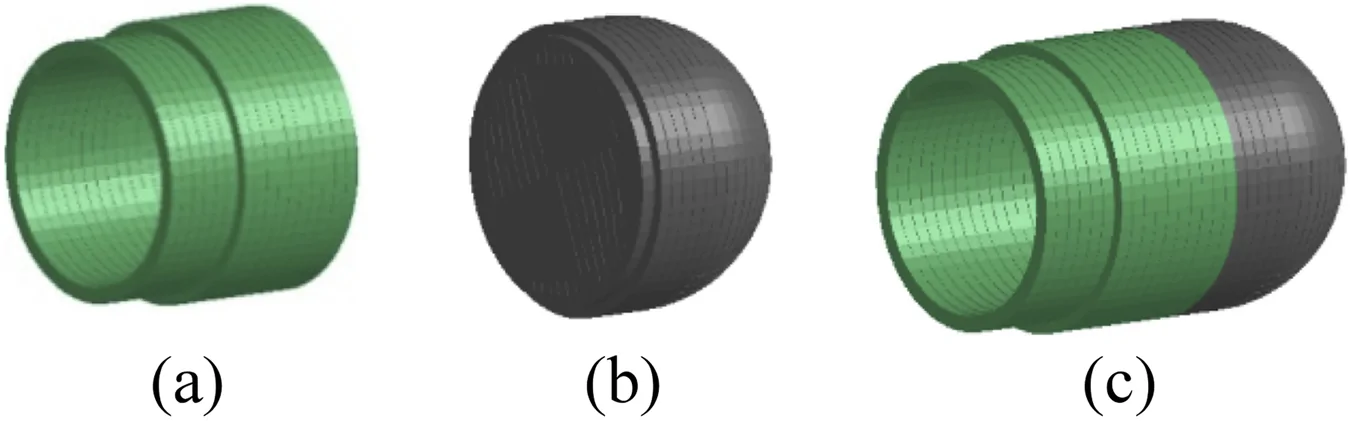
Structural diagram of the SIR-X projectile finite-element model. **(a)** Body; **(b)** nose; **(c)** projectile.

Based on the definitions of the material parameters of the thoracic digital model and the SIR-X projectile finite-element model in Zhang et al. (2025b), Xiao et al. (2025), the material parameters of the digital test projectile and the digital thoracic target were defined, as listed in Table 4.

**TABLE 4:** Material parameters for digital test projectiles and digital thoracic targets.

Category	Component	Density (g/cm3)	Young’s modulus (MPa)	Elastic bulk modulus (MPa)	Poisson’s ratio	Short-term shear modulus (MPa)	Long-term shear modulus (MPa)	Decay constant (m/s)
Thorax	Skin	1.30	31.5	​	0.45	​	​	​
	Rib	1.08	5,500	​	0.20	​	​	​
	Costal cartilage	1.00	49	​	0.40	​	​	​
	Sternum	1.25	250	​	0.25	​	​	​
	Diaphragm	1.00	65.5	​	0.40	​	​	​
	Thoracic vertebra	1.33	355	​	0.26	​	​	​
	Intervertebral disk	1.50	10	​	0.35	​	​	​
	Ligament	1.10	104	​	0.45	​	​	​
	Muscle	1.12	​	2,900	​	0.2	0.195	0.1
	Heart	1.00	​	744	​	0.067	0.065	0.1
	Lung	0.60	​	744	​	0.067	0.065	0.1
Projectile	Nose	0.231	​	​	0.16	​	​	​
	Body	1.354	3,000	​	0.31	​	​	​

Although thoracic organs exhibit nonlinear, viscoelastic, and strain-rate dependent behavior, elastic material formulations were retained in the present CHEST model because we aimed to evaluate and optimize global thoracic responses, including chest displacement and viscous criteria, rather than to predict organ level injury. In addition, the CHEST model had previously been validated at the whole thorax level under the prescribed blunt impact conditions. Therefore, the organ material parameters were kept unchanged to avoid introducing additional parameter interactions during the sensitivity-based optimization of the costal cartilage modulus. Furthermore, the available experiments did not provide organ-level pressure, strain, or deformation data required to identify and validate more complex constitutive parameters.

### 2.4 Numerical simulation setup for blunt ballistic impact

After the digital thoracic target and the digital test projectile were selected and constructed, finite-element simulations were performed for assessing the impacts of non-lethal kinetic energy projectiles on the thoracic target using LS-DYNA and LS-PrePost. A single-point integration Lagrangian algorithm was used to capture the transient impact response. During the impact, the thoracic target was fixed as a whole. The internal tissues and organs of the digital thoracic target had complex contact interactions. Therefore, automatic single-surface contact (CONTACT_AUTOMATIC_SINGLE_SURFACE) was defined among the thoracic tissues and organs. The static and dynamic friction coefficients were both set to 0.10 to permit relative sliding between adjacent thoracic structures without introducing excessive tangential constraint. The segment-based penalty contact formulation was employed with SOFT = 2 to accommodate the rapid and large deformation occurring during the impact. The local penalty stiffness scale factors were set to SFS = SFM = 1.0, and the global penalty stiffness scale factor in CONTROL_CONTACT was set to SLSFAC = 0.1. These values retained the standard penalty stiffness scaling used in LS-DYNA and were kept constant throughout all simulations. The same definition was also applied between the projectile body and the projectile head to account for their possible self-contact. The contact between the projectile and the thoracic model was defined as a global automatic surface-to-surface contact (CONTACT_AUTOMATIC_SURFACE_TO_SURFACE). The initial projectile velocities were set to 56 m/s and 86.5 m/s. Because the projectile head underwent large compressive deformation during the impact, hourglass control (HOURGLASS) was applied to prevent numerical distortion. The contact time step (CONTACT_TIMESTEP), termination time (*CONTROL_TERMINATION), and output options were then defined. The model was calculated and solved using LS-RUN.

Mesh quality and mesh convergence were assessed before the finite-element model was used for response comparison and parameter optimization. Three mesh densities were established, namely, coarse, medium, and fine meshes. Medium mesh was used in the original CHEST model. The elements in the anterior thoracic impact region, including the sternum, ribs, costal cartilage, adjacent soft tissues, and projectile contact region, were locally coarsened or refined, while the mesh outside this region was largely retained to control the computational cost. The information on meshes of different sizes is presented in Table 5.

**TABLE 5:** Mesh configurations used in the convergence analysis.

Mesh level	Local element size in the impact region (mm)	Shell elements	Solid elements	Total nodes	Relative computational time
Coarse	6	2,615	70,149	86,045	0.67
Medium	4	3,634	135,503	120,084	1.00
Fine	2	5,539	214,607	214,098	3.24

All material properties, contact definitions, boundary conditions, initial velocity, and numerical control parameters were kept unchanged among the three models. The convergence analysis was conducted at an impact velocity of 86.5 m/s because this condition resulted in greater thoracic deformation and represented the more severe loading condition.

Peak chest wall displacement, maximum viscous response, peak contact force, and the time of peak displacement were selected as convergence indicators. The relative difference between the medium and fine meshes was calculated using Equation 2.

$$\delta =\frac{\left|R_{\text{fine}}-R_{\text{medium}}\right|}{R_{\text{fine}}}\times 100\%$$ (2)

where R represents the response quantity. A relative difference below 5% was adopted as the mesh convergence criterion.

In addition to the chest wall displacement and viscous response, the contact force and energy transfer during the impact were obtained to provide further biomechanical interpretation of the numerical results. The resultant contact force was obtained from the contact interface between the projectile and the thoracic target. The contact force-time curve was used to characterize the magnitude and duration of the transient impact load.

Energy transfer was evaluated using the history of the kinetic energy of the projectile and the internal energy of the thoracic target. The energy transferred from the projectile at time t was calculated using Equation 3.

$$E_{\text{tr}}\left(t\right)=E_{k,0}-E_{k}\left(t\right)$$ (3)

where *E*_k,0_ is the initial kinetic energy of the projectile and *E*_k_(t) is its kinetic energy at time t . The increase in the internal energy of the thoracic target was also examined to characterize the energy associated with structural deformation. The term energy transfer was used instead of irreversible energy absorption because part of this energy may be recovered during rebound. The contact force and energy histories were extracted for impact velocities of 56 m/s and 86.5 m/s.

## 3 Results and Discussion

### 3.1 Blunt ballistic impact experimental results and discussion

The impact process recorded by the high-speed camera showed that, at an impact velocity of 56 m/s, the motion of the physical thoracic targets with different simulated ribs could be divided into three stages: compression, rebound, and recovery. During the compression stage, the simulated skin–muscle–fat layer was first struck by the projectile and then moved backward together with the simulated ribs. The simulated thorax was, therefore, compressed as a whole. After the ribs reached the maximum displacement, the simulated ribs and the skin–muscle–fat layer gradually rebounded. After the projectile left the thoracic target, the target returned to its pre-impact state. The processes of a projectile impacting thoracic targets with different simulated ribs at an impact velocity of 56 m/s are shown in Figure 9.

**FIGURE 9:**
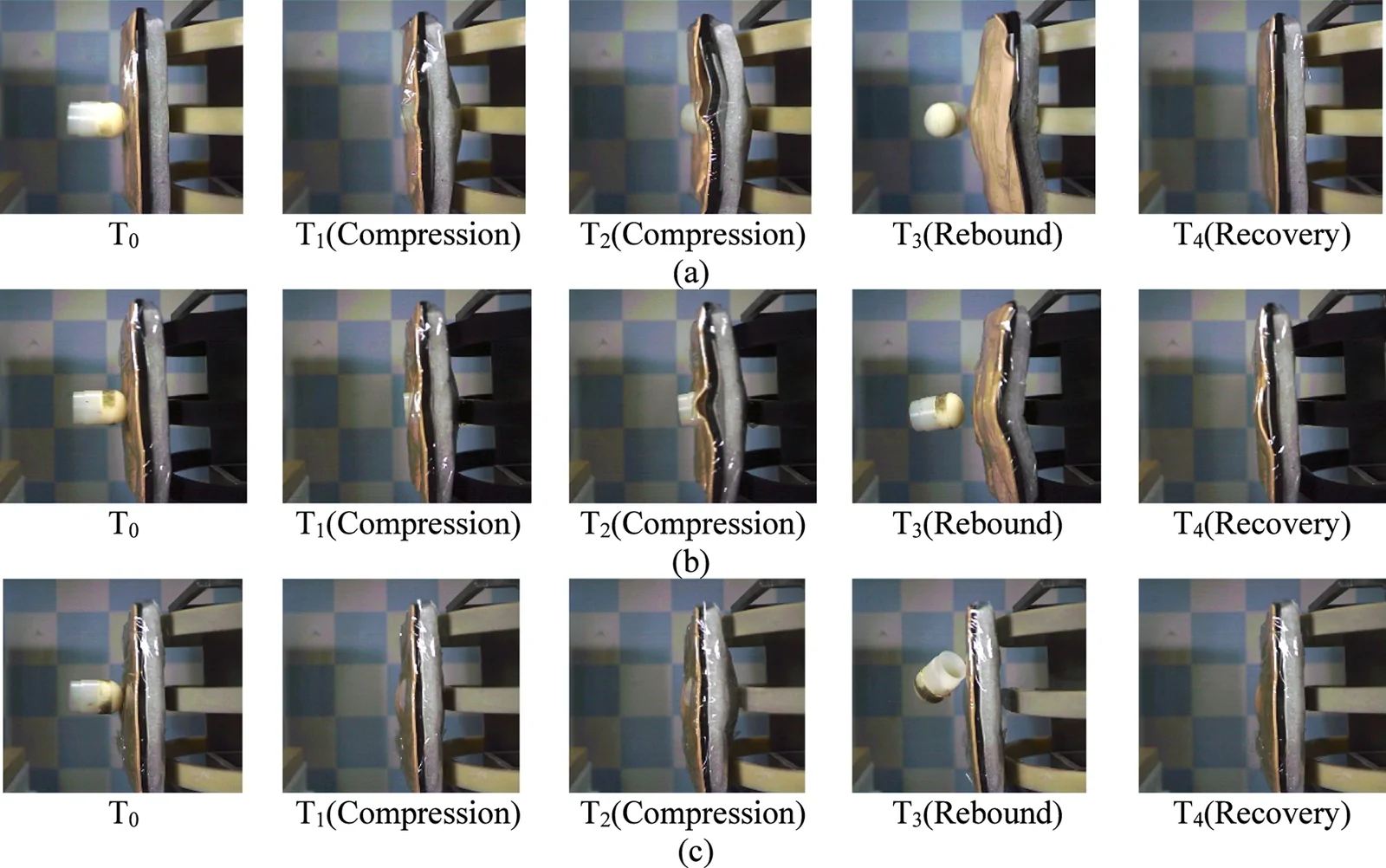
Process of a projectile impacting a thoracic target with different simulated ribs at an impact velocity of 56 m/s (T_0_ = 0 ms, T_1_ = 1.0 ms, T_2_ = 2.0 ms, T_3_ = 3.0 ms, and T_4_ = 4.0 ms). **(a)** Simulated rib 1(PA66+GF15). **(b)** Simulated rib 2(PA66+GF30). **(c)** Simulated rib 3 (GFPA12GF).

Figure 9 illustrates the temporal evolution of the thoracic response during the impact, which can be divided into three stages: compression, rebound, and recovery. T_0_ represents the initial state before substantial thoracic deformation. At T_1_ and T_2_, the projectile remains in contact with the anterior chest wall, and the thoracic displacement progressively increases until maximum compression is reached, corresponding to the compression stage. At T_3_, the stored elastic deformation energy is partially released, causing the anterior chest wall to move toward its initial position while the projectile begins to separate from the thorax (rebound stage). At T_4_, following projectile separation, the contact force decreases to 0, and the thorax enters the recovery stage, during which the thoracic structures gradually return toward their pre-impact configuration through damped deformation and residual energy dissipation. Accordingly, the three stages are explicitly identified in Figure 9 as compression (T_1_–T_2_), rebound (T_3_), and recovery (T_4_).

Comparison of the three stages at an impact velocity of 56 m/s showed that the thoracic targets had the same motion process and no damage occurred in the simulated skin, muscle, fat layers, or simulated ribs. However, visual comparison showed that the compression deformation of the simulated ribs differed after the impact, which was quantified by the magnitude of its rearward displacement following impact. Simulated rib 1 underwent the largest deformation, whereas simulated rib 3 underwent the smallest deformation. This indicates that the simulated rib materials had different mechanical responses under the same impact load.

The thoracic target showed the same compression–rebound–recovery behavior at an impact velocity of 86.5 m/s as that at 56 m/s. The processes of a projectile impacting the thoracic targets with different simulated ribs at a velocity of 86.5 m/s are shown in Figure 10.

**FIGURE 10:**
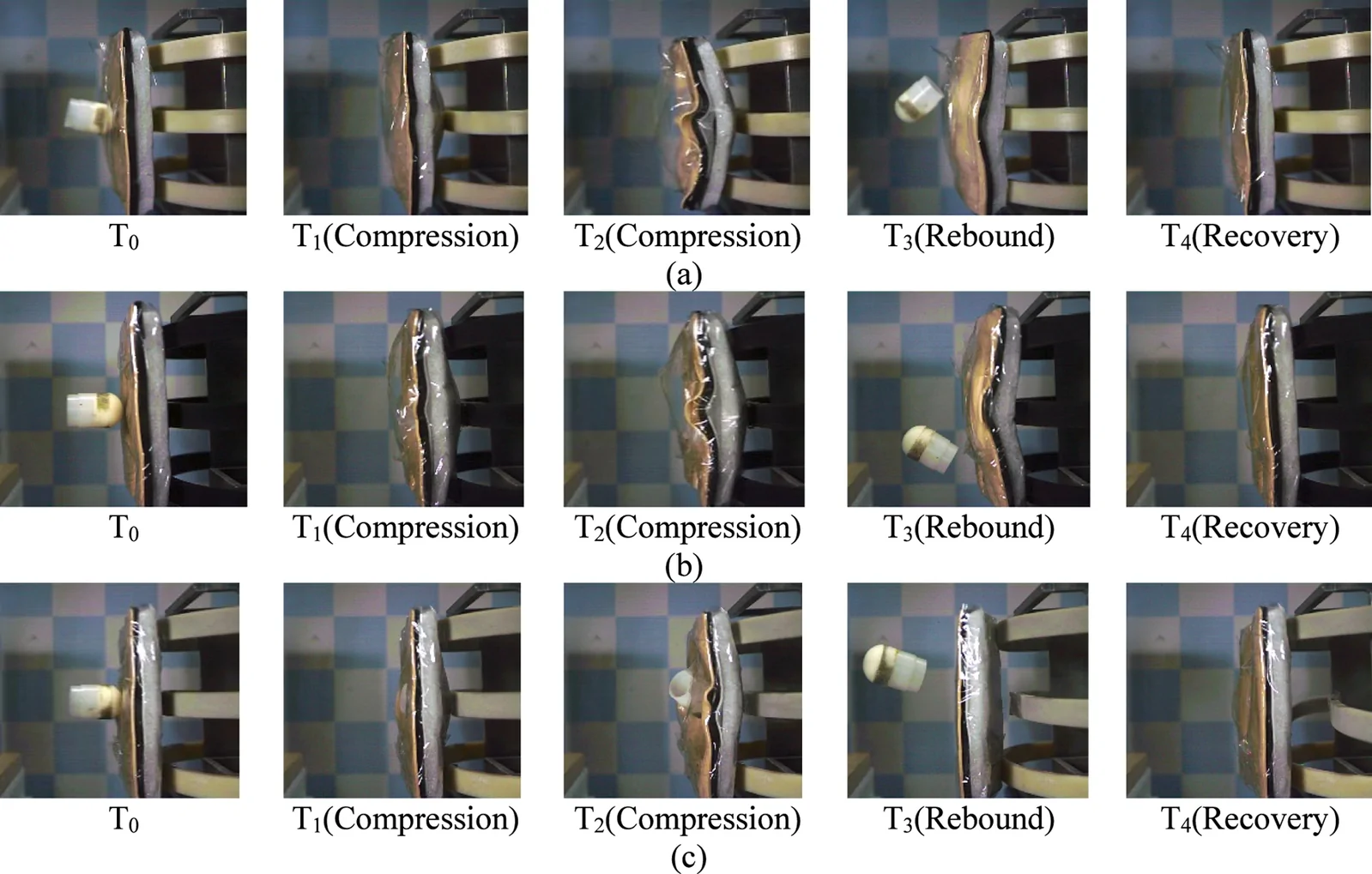
Process of a projectile impacting a thoracic target with different simulated ribs at an impact velocity of 86.5 m/s (T_0_ = 0 ms, T_1_ = 1.0 ms, T_2_ = 2.0 ms, T_3_ = 3.0 ms, and T_4_ = 4.0 ms). **(a)** Simulated rib 1(PA66+GF15). **(b)** Simulated rib 2(PA66+GF30). **(c)** Simulated rib 3 (GFPA12GF).

As shown in Figure 10, the compression deformation refers to the inward displacement of the anterior chest wall relative to its initial undeformed configuration. The deformation occurs primarily around the projectile impact region and develops along the impact direction as the projectile continues to load the thorax. During this process, the localized load is transmitted from the anterior soft tissues to the sternum, costal cartilage, and adjacent ribs, resulting in coordinated deformation of the anterior rib cage. The compression deformation increases until the maximum chest displacement is reached, which represents the maximum compression state of the thoracic target. It should be noted that the maximum compression does not necessarily occur simultaneously with the peak contact force because the thorax may continue to undergo deformation under the combined effects of inertia and structural compliance after the contact force has begun to decrease. After the maximum compression state, the anterior chest wall moves back toward its initial position as the stored deformation energy is partially released, corresponding to the subsequent rebound and recovery stages. Therefore, Figure 10 represents the spatial deformation characteristics of the thorax at the compression stage, particularly the inward displacement of the anterior chest wall and the associated deformation of the sternum–costal cartilage–rib structure.

The results showed that the three targets had the same motion process. However, during rebound, rib fracture occurred in the target with simulated rib 3 in the first test, as shown at T = 4.0 ms. No damage was observed in the simulated skin, muscle, or fat layers. The damage to the thoracic target with simulated rib 3 is shown in Figure 11.

**FIGURE 11:**
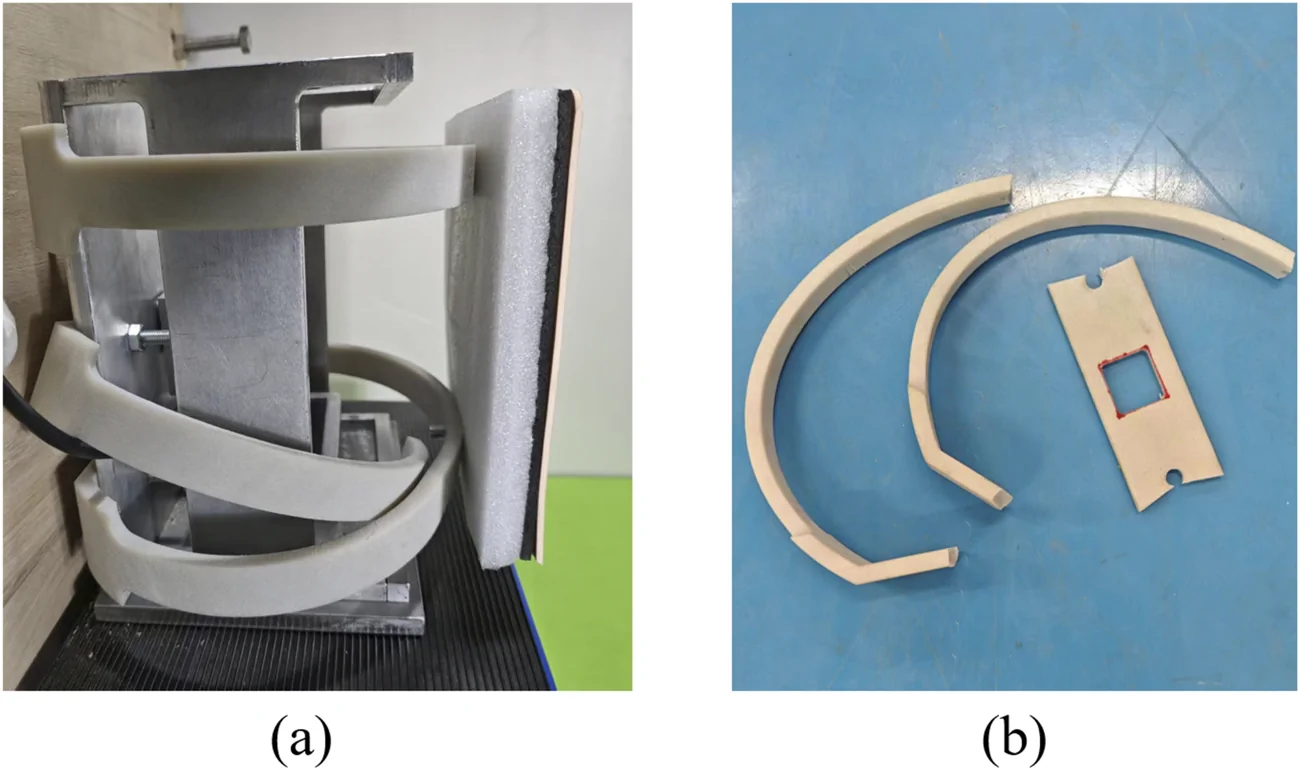
Damage to the thoracic target following the impact of simulated rib 3. **(a)** Thoracic target. **(b)** Simulated rib 3.

After the signals collected by the displacement sensor were transmitted to the data acquisition system, the measured displacement data were filtered using the CFC1000 channel frequency class and then normalized according to AEP-99. The digital filtering algorithm was implemented according to SAE J211 (SAE, 2014) and ISO 648:2015 (ISO, 2015). A fourth-order Butterworth low-pass filter was used for signal processing, and the sampling frequency was specified. The transfer function of the corresponding second-order Butterworth low-pass filter is given in Equation 4.

$$Y\left(t\right)=a_{0}X\left(t\right)+a_{1}X\left(t-1\right)+a_{2}X\left(t-2\right)+b_{1}Y\left(t-1\right)+b_{2}Y\left(t-2\right)$$ (4)

Here, $X\left(t\right)$ and $Y\left(t\right)$ are the input and output values, respectively. $a_{0}$ , $a_{1}$ , and $a_{2}$ , are the feedforward coefficients, whereas *b*_*1*_ and *b*_*2*_ are the recursive feedback coefficients of the second-order Butterworth filter. Specifically, *b*_*2*_ is associated with the previous output term *Y(t-2).* The coefficients are calculated using Equation 5.

$$\left\{\begin{array}{l} w_{d}=2\pi \times \text{CFC}\times 2.0775\\ w_{a}=\sin\left(w_{d}\times T/2\right)/\cos\left(w_{d}\times T/2\right)\\ a_{0}=w_{a}^{2}/\left(1+\sqrt{2}\times w_{a}+w_{a}^{2}\right)\\ a_{1}=2\times a_{0}\\ a_{2}=a_{0}\\ b_{1}=-2\times \left(w_{a}^{2}-1\right)/\left(1+\sqrt{2}\times w_{a}+w_{a}^{2}\right)\\ b_{2}=\left(-1+\sqrt{2}\times w_{a}-w_{a}^{2}\right)/\left(1+\sqrt{2}\times w_{a}+w_{a}^{2}\right) \end{array}\right.$$ (5)

To achieve fourth-order Butterworth low-pass filtering, the raw data were passed through the filter twice. The filtered data were then normalized. The normalization was based on the 50th-percentile male chest depth of 236 mm determined in the PMHS impact tests by Bir (2000), which has been used as a standard biomechanical reference in blunt impact tests. The thoracic dimension of the physical thoracic target was normalized by a scaling factor. The chest depth of the physical target was 209 mm, giving a depth scaling factor λ of 1.13. The normalized displacement was obtained by multiplying the measured displacement by λ . The normalized velocity was calculated using Equation 6, where $Y\left(x\right)$ is the displacement at time x , and $Y^{\prime }\left(x\right)$ is the instantaneous velocity at time x .

$$Y^{\prime }\left(x\right)=\frac{dY\left(x\right)}{dx}$$ (6)

Based on the filtered and normalized displacement–time data, the displacement responses of the physical thoracic targets were evaluated against the AEP-99 displacement criterion. The normalized displacement–time histories of the targets fitted with the three simulated rib materials were compared with the prescribed displacement corridors at impact velocities of 56 m/s and 86.5 m/s, as shown in Figure 12. The upper and lower black curves define the allowable response corridor, while the colored curves represent the measured responses of the targets with simulated ribs 1, 2, and 3. The comparison focuses on the evolution of chest-wall displacement during the first 2 ms after impact, including the onset and rate of compression, displacement magnitude, and whether each response remains within the prescribed corridor. This comparison provides the basis for evaluating the influence of rib material on the thoracic displacement response and for selecting the physical target configuration that satisfies the displacement-based validation criterion.

**FIGURE 12:**
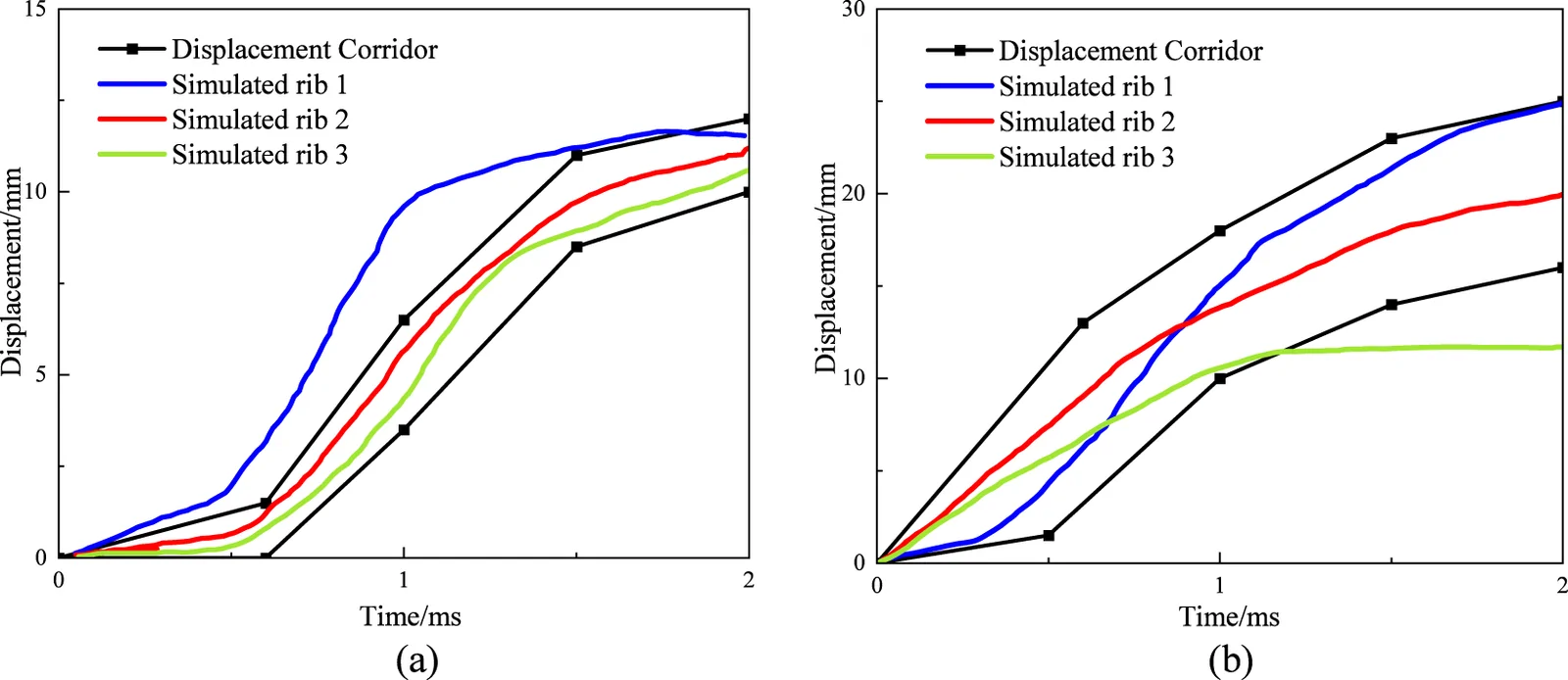
Displacement–time curve for a physical thoracic target subjected to projectile impact. **(a)** Impact velocity: 56 m/s. **(b)** Impact velocity: 86.5 m/s.

As shown in Figure 12, when the projectile impact velocity was 56 m/s, the displacement–time curves of the physical thoracic targets with simulated rib 2 and simulated rib 3 were within the corridor specified in AEP-99. In contrast, the curve of the target with simulated rib 1 exceeded the upper corridor limit during the first 2 ms. When the impact velocity was 86.5 m/s, the curves of the targets with simulated rib 1 and simulated rib 2 were within the standard corridor. The curve of the target with simulated rib 3 remained within the corridor during the first 1 ms. However, owing to rib fracture, its displacement–time curve no longer increased and fell below the lower corridor limit from 1 to 2 ms. According to the standard requirements, the physical thoracic target with simulated rib 2 met the validation criteria.

From the perspective of structural mechanics, the resistance of a rib to bending is related to its flexural rigidity, *EI*, where *E* is Young’s modulus and *I* is the second moment of area. When the rib geometry, boundary conditions, and loading conditions remain unchanged, an increase in Young’s modulus generally increases structural stiffness and reduces the displacement response before material damage occurs.

However, the three candidate rib materials used in the present experiments differed not only in Young’s modulus but also in density, Poisson’s ratio, glass fiber content, manufacturing process, and failure behavior. Therefore, the experimental results cannot be interpreted as a strictly single-parameter relationship between Young’s modulus and displacement. At an impact velocity of 56 m/s, the target fitted with PA66+GF15 ribs, with a Young’s modulus of 15.0 GPa, showed the largest displacement and exceeded the upper boundary of the AEP-99 corridor. The PA66+GF30 and GFPA12GF targets, with Young’s moduli of 19.3 and 12.9 GPa, respectively, remained within the displacement corridor.

At an impact velocity of 86.5 m/s, the PA66+GF30 target maintained structural integrity, and its displacement response remained within the prescribed corridor. In contrast, the GFPA12GF rib underwent fracture during impact. The subsequent reduction in its displacement response resulted from the loss of structural continuity and load transfer rather than from increased rib stiffness. These results indicate that Young’s modulus influences the displacement response before structural failure, but impact durability, material toughness, and manufacturing characteristics also have important effects. Among the three materials, PA66+GF30 provided the most appropriate balance between stiffness, structural integrity, and displacement response under the investigated impact conditions.

The results do not show a simple monotonic relationship between the nominal Young’s modulus and the measured displacement because all other properties of the tested materials were not identical. Nevertheless, the excessive displacement of the PA66+GF15 target and the fracture of the GFPA12GF target demonstrate that both insufficient stiffness and inadequate impact durability can prevent a surrogate rib from satisfying the prescribed response requirements. The PA66+GF30 ribs provided a stable displacement response without structural failure at both impact velocities.

After the displacement–time curves of the thoracic targets were obtained, the viscous response VC during the impact was calculated. The VC is an important index for evaluating blunt thoracic injury. It was first proposed by Lau and Viano (1986). This criterion was initially used to assess blunt injury caused by vehicle crashes (Viano, 1989). It was later applied to blunt-impact studies (Bir and Viano, 2004) and incorporated into AEP-99 to verify whether thoracic targets meet the standard requirements. The calculation equations are as follows:

$${VC}_{\max}={\left(V\left(t\right)C\left(t\right)\right)}_{\max}$$ (7)

$$V\left(t\right)\cdot C\left(t\right)=\frac{dD\left(t\right)}{dt}\cdot \frac{D\left(t\right)}{D_{0}}$$ (8)

In Equation 7, VC is the viscous response, VC_max_ is the peak viscous response, $V\left(t\right)$ is the instantaneous thoracic compression velocity, and $C\left(t\right)$ is the thoracic compression ratio. In Equation 8, $D_{0}$ is the initial external thoracic thickness and $D\left(t\right)$ is the instantaneous thoracic thickness. Here, $D_{0}=0.236$ m, which represents the chest depth of a 50th-percentile man. Based on the displacement–time data at impact velocities of 56 m/s and 86.5 m/s and the VC equations, the time-varying VC values were calculated. The corresponding VC –time curves are shown in Figure 13.

**FIGURE 13:**
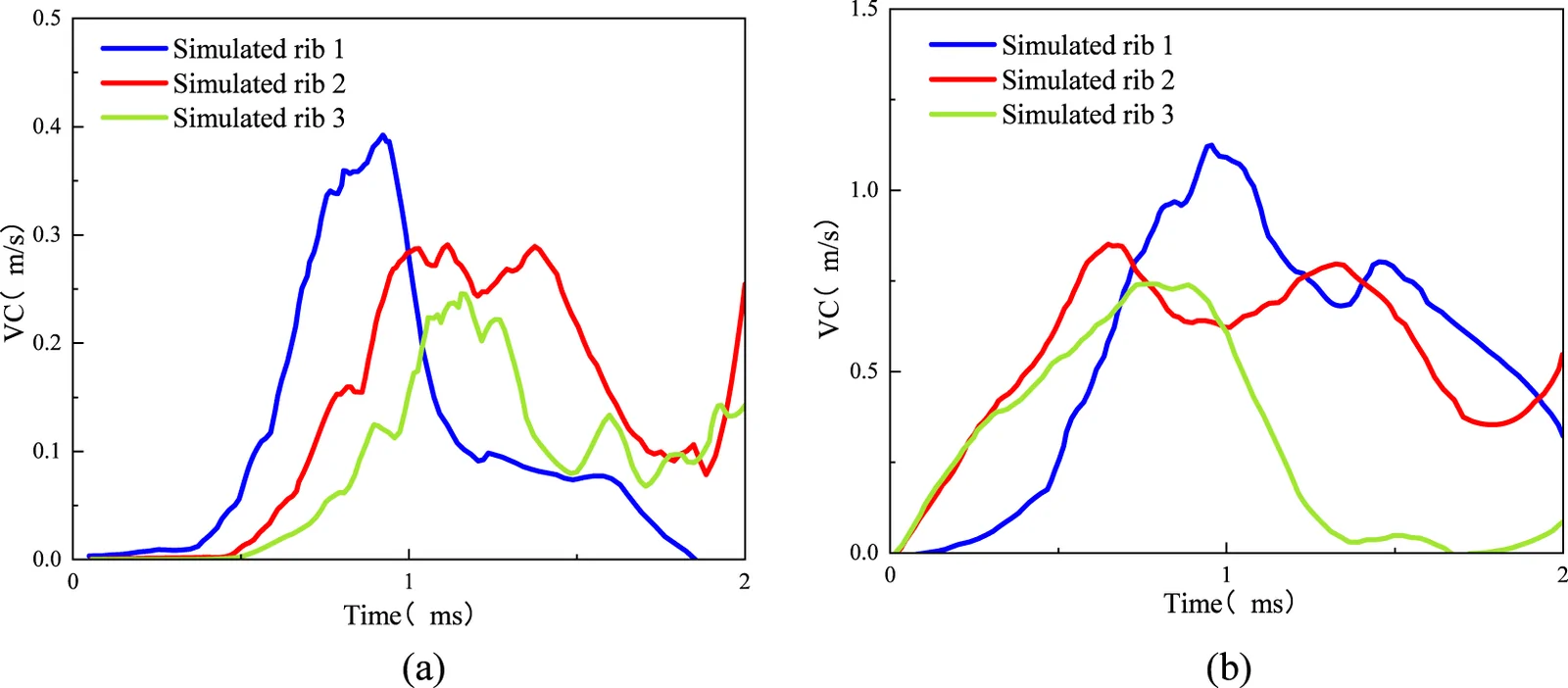
VC-time curve for a physical thoracic target subjected to projectile impact. **(a)** Impact velocity: 56 m/s. **(b)** Impact velocity: 86.5 m/s.

The VC_max_ value specified in AEP-99 corresponds to the peak of each curve. At both impact velocities, the thoracic target with simulated rib 1 had the largest VC_max_, whereas the target with simulated rib 3 had the smallest VC_max_. The VC_max_ values of the targets with the three simulated ribs were summarized and compared with the VC_max_ ranges specified in AEP-99, as shown in Table 6.

**TABLE 6:** VC_max_ and its standard range for thoracic targets fitted with three types of simulated ribs (unit: m/s).

Impact velocity (m/s)	Simulated rib			Standard range
	1	2	3	
56	0.392	0.291	0.246	0.28-0.32
86.5	1.125	0.841	0.732	0.78-0.85

The VC_max_ values of the target with simulated rib 2 were within the AEP-99’s prescribed ranges, whereas those of the targets with simulated ribs 1 and 3 were outside the required ranges. Combined with the displacement–time results, these findings confirmed that the target with simulated rib 2 satisfied the AEP-99 validation criteria and was, therefore, selected for comparison of the subsequent physical–digital response.

In addition, Ma et al. (2022) reported that the longitudinal Young’s modulus of human cortical bone is usually approximately 15–25 GPa and that the material properties of human rib cortical bone are strongly affected by age. According to an age-related regression model based on a large dataset of rib cortical bone, the elastic modulus of rib cortical bone in typical adult men falls within approximately 13–21 GPa. Therefore, the Young’s modulus of simulated rib 2, 19.3 GPa, lies within the reasonable range reported for human rib cortical bone and is at the medium-to-high level relative to the age-related mean for adult men. This indicates good biofidelity. The Young’s modulus of simulated rib 2 (19.3 GPa) also falls within the reported range for adult human rib cortical bone, providing additional support for its biomechanical relevance.

After the conclusions were drawn, the variability in the experimental data was statistically evaluated. The repeatability of the physical impact tests was evaluated using the results of the three repeated tests conducted for each condition. The peak normalized displacement and VC_max_ are reported as mean and standard deviation in Table 7. The coefficient of variation was used as an additional indicator of result dispersion.

**TABLE 7:** Statistical results of the repeated physical impact tests.

Impact velocity (m/s)	Rib material	Actual impact velocity, mean ± SD (m/s)	Peak displacement, mean ± SD (mm)	CV of peak displacement (%)	VC_max_, mean ± SD (m/s)	CV of VC_max_ (%)
56.0	PA66+GF15	56.0 ± 1.76	11.4 ± 0.53	4.65	0.392 ± 0.04	10.20
56.0	PA66+GF30	56.0 ± 1.41	12.3 ± 0.61	4.96	0.291 ± 0.03	10.31
56.0	GFPA12GF	56.0 ± 2.05	11.7 ± 0.32	2.74	0.246 ± 0.03	12.20
86.5	PA66+GF15	86.5 ± 1.03	24.9 ± 1.63	6.55	1.125 ± 0.12	10.67
86.5	PA66+GF30	86.5 ± 1.65	18.5 ± 1.42	7.68	0.841 ± 0.09	10.70
86.5	GFPA12GF	86.5 ± 1.99	12.6	—	0.732	—

Except for the GFPA12GF condition at 86.5 m/s, each statistical value was based on three repeated tests. SD denotes the sample standard deviation, and CV denotes the coefficient of variation.

Because the simulated rib made of GFPA12GF fractured at an impact velocity of 86.5 m/s in the first test, statistical values for SD and CV could not be obtained. The coefficients of variation for peak displacement ranged from 2.74% to 7.68%, while those for VC_max_ ranged from 10.20% to 12.20%. These results indicate acceptable repeatability under the investigated impact conditions. The statistical variation was also considered when determining whether the responses satisfied the AEP-99 acceptance ranges.

### 3.2 Results and discussion of numerical simulation of blunt ballistic impact

LS-PREPOST was used to load the impact process of the digital test projectile on the digital thoracic target and to export the images and numerical results. The impact process of the SIR-X digital projectile on the digital thoracic target is shown in Figure 14.

**FIGURE 14:**
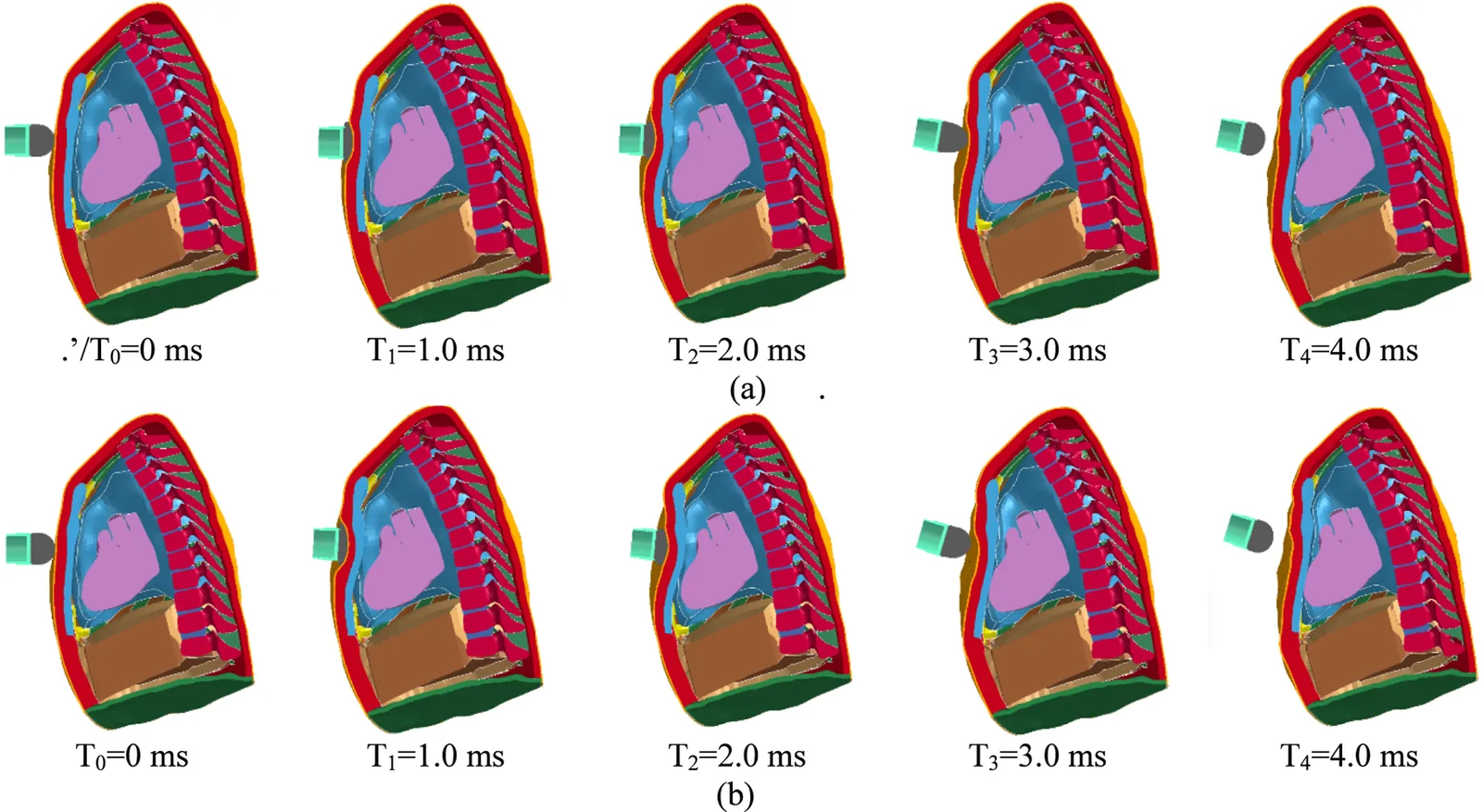
Process of a projectile impacting the digital thoracic target (cross-section). **(a)** Impact velocity: 56 m/s. **(b)** Impact velocity: 86.5 m/s.

The mesh convergence results are summarized in Table 8. Refining the mesh from the coarse level to the medium level resulted in noticeable changes in the principal response quantities. In contrast, the differences between the medium and fine meshes were 4.34% for peak chest wall displacement, 2.73% for VC_max_, 3.90% for peak contact force, and 4.07% for the time of peak displacement. All differences were below the adopted convergence criterion of 5%.

**TABLE 8:** Comparison of biomechanical responses for different mesh densities.

Mesh level	Peak displacement (mm)	VC_max_	Peak contact force (kN)	Time of peak displacement (ms)
Coarse	21.4	0.956	25.891	2.41
Medium	17.2	0.821	23.546	2.79
Fine	16.5	0.799	22.605	2.88
Difference between medium and fine meshes (%)	4.34	2.73	3.90	4.07

These results indicate that further mesh refinement produced only limited changes in the principal biomechanical responses. The medium mesh was, therefore, considered sufficiently converged with regard to the objectives of this study. It was retained for the subsequent physical and finite-element response comparison and parameter optimization because it provided an appropriate balance between numerical accuracy and computational efficiency.

After the simulations were completed, the chest-wall displacement data were extracted. The signals were processed using the same fourth-order Butterworth low-pass filter as that used in the physical tests. The displacement–time curves were then plotted. The chest VC values were calculated using Equations 7, 8, and the VC–time curves were plotted. The curve results are shown in Figure 15.

**FIGURE 15:**
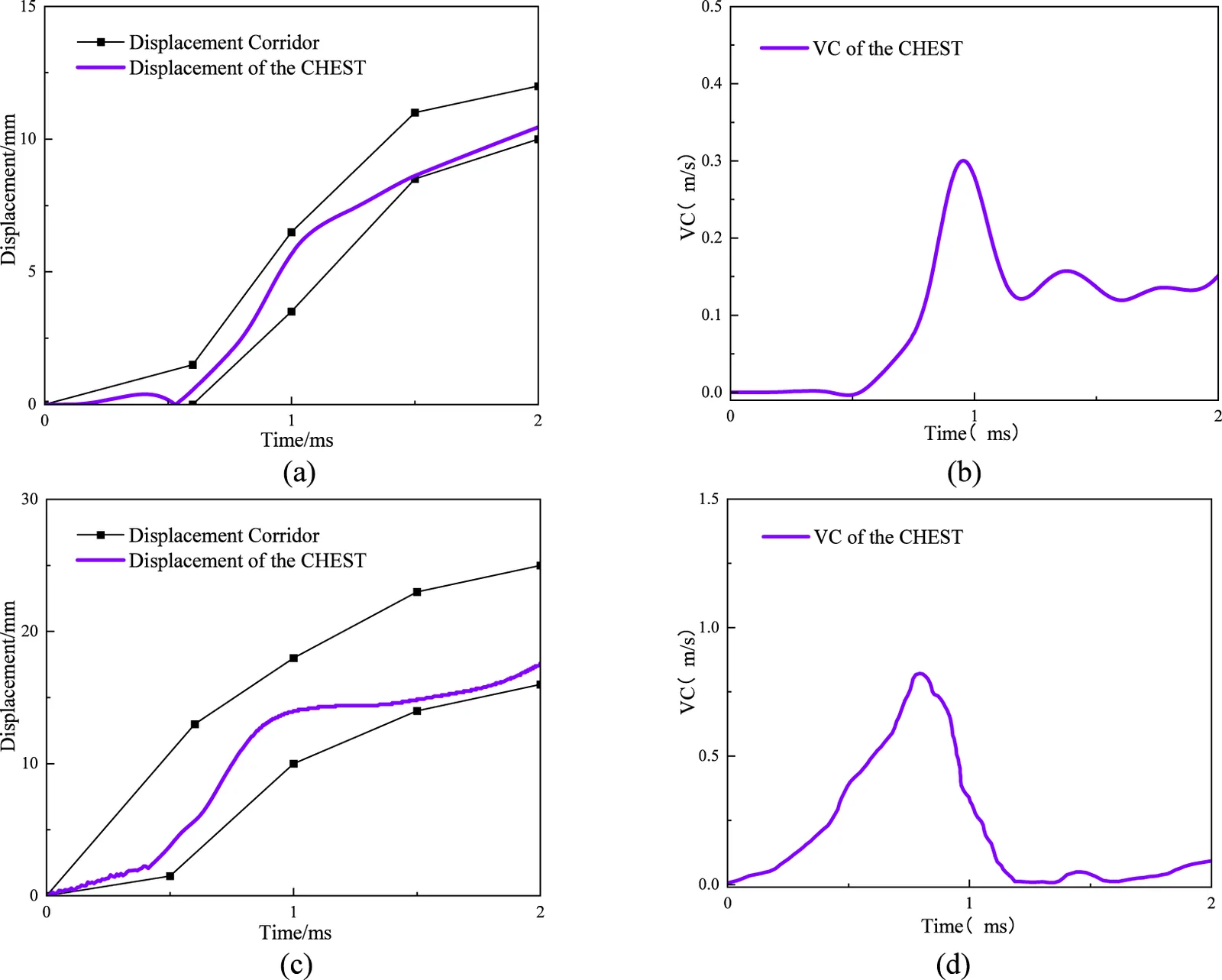
Displacement–time curve and VC-time curve of the CHEST. **(a)** Displacement–time curve of the CHEST (56 m/s). **(b)** VC-time curve of the CHEST (56 m/s). **(c)** Displacement–time curve of the CHEST (86.5 m/s). **(d)** VC-time curve of the CHEST (86.5 m/s).

At both impact velocities, the CHEST chest-wall displacement–time curves remained within the AEP-99 corridors, and the corresponding VCmax values of 0.319 and 0.821 m/s were also within the prescribed ranges. Therefore, the CHEST model satisfies the AEP-99 validation criteria under both impact velocities.

To visually analyze the stress response of the skeleton in the CHEST digital thoracic target, equivalent stress contours of the skeleton during the impact were generated, as shown in Figure 16.

**FIGURE 16:**
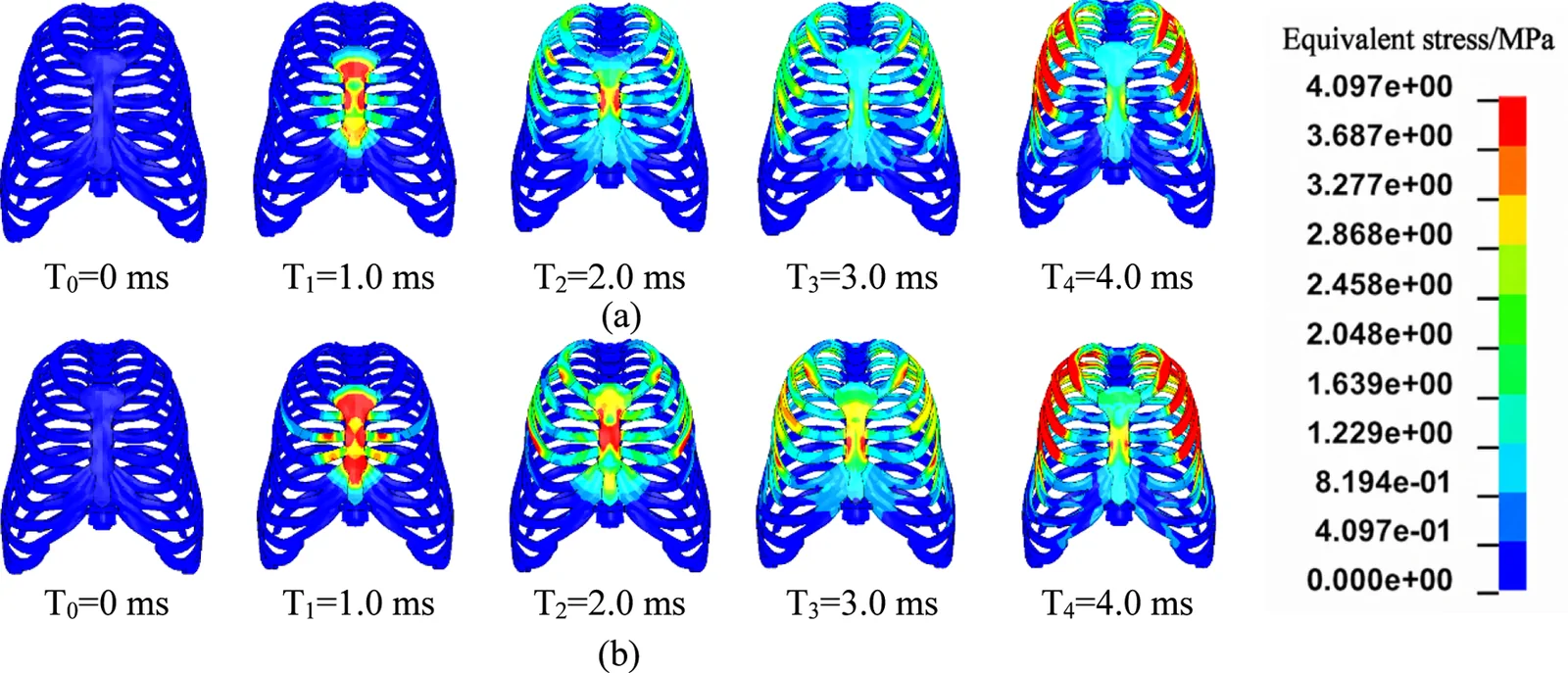
Equivalent stress contours of the skeleton. **(a)** Impact velocity: 56 m/s. **(b)** Impact velocity: 86.5 m/s.

After projectile impact, the impact pressure was transmitted from the chest surface to the skeleton. The skeletal stress then began to increase. At 1 ms, the stress was mainly concentrated in the sternum and adjacent costal cartilage. At 2 ms, the stress spread to the ribs. At 3 ms, it further propagated to the ribs on both sides. At 4 ms, the stress concentration had transferred from the sternum to the bilateral rib regions. The change in the skeletal stress concentration region reflects multiple reflections and transmission of the stress wave. Under the impact load, high stress first appeared in the sternum and ribs near the center of the impact. The stress then rapidly spread outward along the ribs. As the impact velocity increased, the peak stress increased and the wave propagation range expanded more rapidly. The high-stress region extended more clearly from the sternum to the ribs and the outer thoracic cage. The overall stress distribution showed clear temporal and spatial gradients, indicating that the thoracic structure has distinct stress wave transmission and attenuation characteristics. The simulated stress wave propagation characteristics of the thoracic skeleton are consistent with the simulation results in Xiao et al. (2025). Based on the results of the stress contour analysis of the digital thoracic skeleton, future studies can further assess the risk of fracture injury under impacts from non-lethal kinetic energy projectiles to the chest.

It should be noted that stress wave attenuation does not necessarily imply a monotonic decrease in the instantaneous peak stress of the entire thoracic skeleton. From $T_{1}$ to $T_{3}$ , the initial impact load was progressively distributed from the sternum and adjacent costal cartilage to a wider region of the ribs. This spatial redistribution and wave dispersion reduced the local stress concentration near the impact center. At $T_{4}$ , as the thoracic deformation continued to develop, the bilateral ribs underwent more pronounced bending and load transfer. In addition, the transmitted stress waves may have been reflected at structural junctions and constrained regions, including the sternocostal and posterior rib connections. The superposition of the transmitted and reflected waves, therefore, resulted in a transient increase in the local stress within the bilateral rib regions. Thus, the increase observed at $T_{4}$ represents a secondary spatial concentration of skeletal stress rather than a reversal of the overall stress wave attenuation process.

The contact force time curves at the two impact velocities are shown in Figure 17. After the projectile made contact with the anterior thoracic wall, the contact force increased rapidly, reached a peak, and subsequently decreased to 0 as the projectile rebounded and separated from the thorax. The peak contact forces were 19,364 N and 23,546 N at 56 m/s and 86.5 m/s, respectively. The higher impact velocity produced a larger peak contact force, indicating a greater transient load applied to the anterior thoracic structure.

**FIGURE 17:**
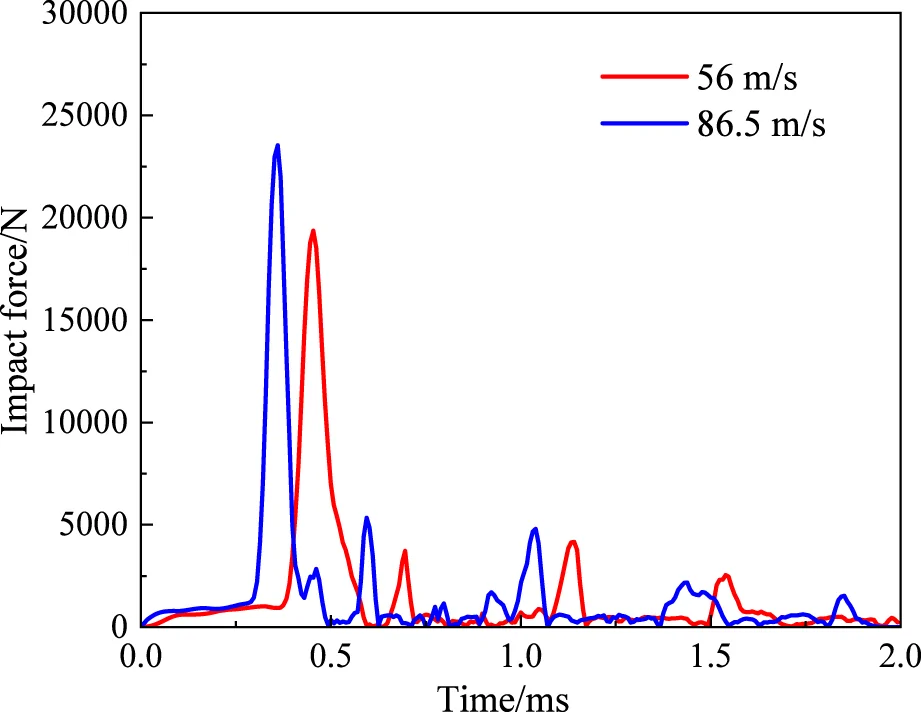
Impact force time curves between the projectile and the CHEST thoracic model at impact velocities of 56 m/s and 86.5 m/s.

The energy transfer histories are presented in Figure 18. During contact, the kinetic energy of the projectile decreased, while the internal energy of the thoracic target increased as a result of deformation of the chest wall and internal thoracic structures. The maximum thoracic internal energies were 27.69 J at 56 m/s and 59.48 J at 86.5 m/s. The larger energy transfer at 86.5 m/s was consistent with the greater chest wall displacement and VC_max_ obtained under the higher velocity condition.

**FIGURE 18:**
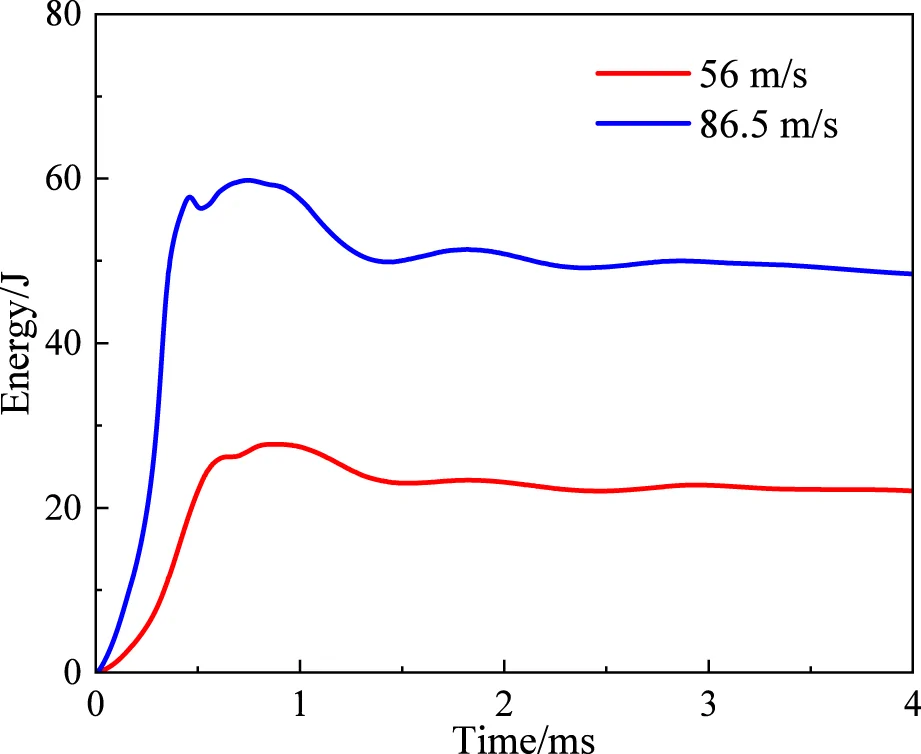
Energy transfer time curves from the projectile to the CHEST thoracic model at impact velocities of 56 m/s and 86.5 m/s.

The contact force and energy transfer results provide complementary descriptions of the impact process. The contact force characterizes the transient loading applied by the projectile, whereas the energy history describes the transfer of projectile kinetic energy into thoracic deformation. These results strengthen the biomechanical interpretation of the numerical response without changing the primary validation criteria based on chest wall displacement and VC_max_.

The thoracic impact response was further interpreted by combining the displacement, projectile–thorax contact force, and thoracic internal energy histories. These three responses describe different but closely related aspects of the impact process. The contact force represents the external transient load applied by the projectile, the displacement represents the global deformation of the anterior chest wall, and the thoracic internal energy reflects the energy associated with deformation and material dissipation within the thoracic structures.

At the beginning of impact, the projectile contacted the anterior chest wall, and the contact force increased rapidly. This stage corresponded to the rapid deceleration of the projectile and the initial compression of the skin, soft tissues, sternum, costal cartilage, and ribs. The thoracic displacement continued to increase as the impact load was transferred from the anterior contact region to the surrounding thoracic structures. The peak contact force occurred at approximately 0.5 ms, whereas the maximum chest displacement occurred at approximately 2.0 ms. The difference between these two time points indicates that the thorax continued to undergo deformation because of its inertia and structural compliance even after the contact force had begun to decrease.

During the compression stage, the kinetic energy transferred from the projectile was converted into thoracic kinetic energy, deformation energy, and material dissipation. Accordingly, the thoracic internal energy increased rapidly during the period of positive contact force. After the projectile rebounded and separated from the thorax, the contact force decreased to 0. The subsequent decrease in chest displacement represented elastic recovery of the thoracic structures. A slight reduction in internal energy observed during the rebound indicates that part of the stored deformation energy was returned through structural recovery. The remaining internal energy at the end of the simulation represented energy retained through deformation, damping, contact, and other dissipative mechanisms.

These results demonstrate that the maximum contact force and the maximum displacement describe different stages of the impact response. The contact force primarily characterizes the intensity of the initial loading, whereas the maximum displacement reflects the combined effects of loading, thoracic stiffness, inertia, and structural deformation. Therefore, a larger contact force does not necessarily correspond to a proportionally larger displacement. The displacement response depends not only on the magnitude of the applied force but also on the duration of loading and the effective stiffness of the thoracic structure.

When the impact velocity increased from 56 m/s to 86.5 m/s, the initial kinetic energy of the projectile increased substantially because kinetic energy is proportional to the square of velocity. Consequently, the higher velocity condition produced a greater transient contact load and transferred more energy to the thoracic target.

At 86.5 m/s, the peak contact force increased from 19,364 N to 23,546 N, while the maximum thoracic internal energy increased from 27.69 J to 59.48 J. The increase in the transferred energy produced greater compression of the anterior chest wall and resulted in a larger displacement and viscous response. Thus, the larger displacement at 86.5 m/s was not simply caused by the higher velocity itself but by the combined effects of increased projectile kinetic energy, greater contact loading, and greater energy transfer into the deformable thoracic structures.

At 56 m/s, the lower kinetic energy resulted in a smaller contact force and less energy transfer. The thoracic structure, therefore, experienced less global compression. In contrast, the higher velocity condition increased both the loading intensity and the amount of energy involved in thoracic deformation, thereby increasing the mechanical severity of the impact.

The temporal relationships among contact force, thoracic displacement, and internal energy provide a more complete description of the impact mechanism. The contact force increased first and represented the transfer of momentum from the projectile to the thorax. The displacement then increased as the anterior chest wall and surrounding structures underwent deformation under the applied load. At the same time, the thoracic internal energy accumulated as the projectile kinetic energy was transferred into structural deformation and material dissipation. After projectile separation, the contact force returned to 0, while the thorax continued to undergo deformation and recovery because of inertia and elasticity.

Therefore, the contact force curve describes the external loading process, the displacement curve describes the global structural response, and the internal energy curve describes the energy conversion process. Their combined interpretation provides a more physically meaningful assessment of thoracic impact behavior than consideration of the individual response trends alone.

From a biomechanical perspective, the anterior impact load was initially applied to the skin and soft tissues and was subsequently transferred to the sternum, costal cartilage, and adjacent ribs. The costal cartilage and ribs contributed to effective compliance of the anterior thoracic wall. Their deformation allowed the impact load to be distributed from the localized contact region to a wider area of the rib cage.

A more compliant thoracic structure generally permits greater displacement and longer deformation, whereas a stiffer structure may reduce displacement but produce a higher transient contact force. Therefore, the displacement and contact force responses must be interpreted together. Excessive compliance may result in large chest compression, while excessive stiffness may increase the magnitude of the transmitted force. The validated thoracic target should, therefore, reproduce an appropriate balance between force transmission, structural deformation, and energy transfer.

## 4 Optimization and application

### 4.1 Comparison of Impact responses between physical and digital thoracic targets

According to the AEP-99 impact conditions at projectile velocities of 56 m/s and 86.5 m/s, both the physical and numerical impact tests showed that the displacement–time curves of the 3-rib thoracic model and the digital thoracic model were within the specified corridor. Their VC_max_ values also met the specified range. These results verified that both the physical and digital thoracic targets satisfied the standard requirements. However, the chest-wall displacement–time curves showed differences in their impact responses. Therefore, the impact responses of the two targets were compared. The comparisons of the displacement–time curve between the physical and digital thoracic targets at impact velocities of 56 m/s and 86.5 m/s are shown in Figure 19.

**FIGURE 19:**
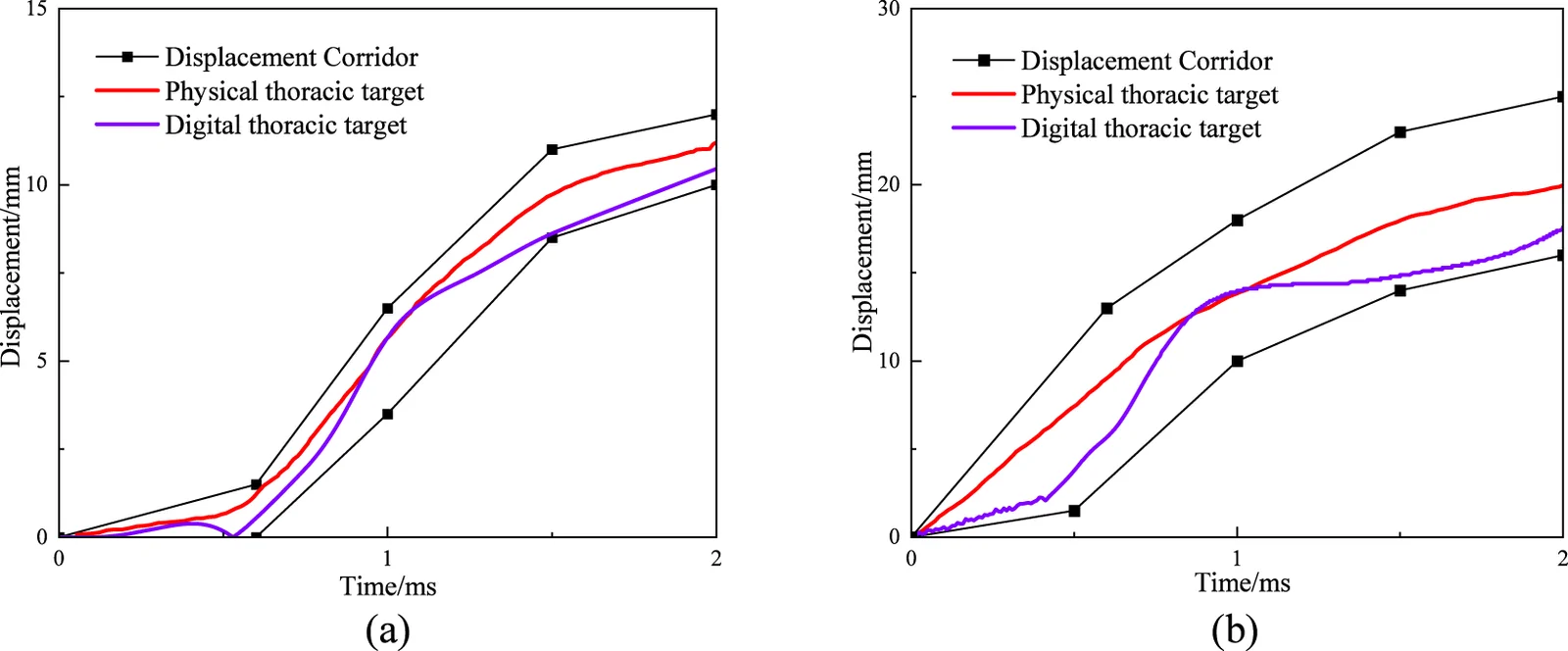
Comparison of displacement–time curves between physical and digital thoracic targets upon impact. **(a)** Impact velocity: 56 m/s. **(b)** Impact velocity: 86.5 m/s.

At both impact velocities, the displacement–time curves of the physical and digital thoracic targets remained within the prescribed corridor, satisfying the standard validation requirements. At 56 m/s, the two responses were similar during the first 1 ms, with the difference increasing gradually between 1 and 2 ms. At 86.5 m/s, the chest-wall displacements were comparable at 1 ms, although larger discrepancies were observed during both the intervals of 0–1 ms and 1–2 ms. To quantify the numerical difference between the two curves and eliminate the effects of data dimensions and value ranges on error evaluation, the normalized mean absolute error (NMAE) was used to characterize the curve difference (De la Calle-Arroyo et al., 2025). NMAE is defined as the ratio of the mean absolute error to the range of the reference data. A smaller NMAE indicates a stronger agreement between the two curves. NMAE is calculated as follows.

$$\text{NMAE}=\frac{\text{MAE}}{y_{\max}-y_{\min}}$$ (9)

Here, MAE stands for mean absolute error and is calculated as follows:

$$\text{MAE}=\frac{1}{n}\sum_{i=1}^{n}\;\left|y_{i}-{\hat{y}}_{i}\right|$$ (10)

In Equation 9, *y*_*max*_ is the maximum value from the physical test and *y*_*min*_ is the minimum value from the physical test. In Equation 10, *n* is the total number of curve points. *y*_*i*_ denotes the displacement–time data for the projectile’s impact on the physical thoracic target, and ${\hat{y}}_{i}$ denotes the displacement–time data for the projectile’s impact on the digital thoracic target.

Because the two curves had different sampling frequencies, they were first resampled to the same number of equally spaced data points by linear interpolation. The MAE was then calculated from the interpolated data to quantitatively evaluate the average numerical deviation between the two curves. The calculated MAE and NMAE between the simulation and physical tests at impact velocities of 56 m/s and 86.5 m/s are listed in Table 9.

**TABLE 9:** MAE and NMAE for the displacement of digital and physical thoracic targets under impact.

Projectile impact velocity (m/s)	MAE (mm)	NMAE (%)
56	0.548	4.8
86.5	2.194	12.5

### 4.2 Digital thoracic target parameter tuning and optimization based on the impact response of a physical thoracic target

The differences in the impact response between the digital and physical thoracic targets were examined at the two projectile impact velocities. The NMAE was 4.8% at 56 m/s and 12.5% at 86.5 m/s. Based on the simulation–experiment error level reported in De la Calle-Arroyo et al. (2025), an error threshold of 5% was adopted. Therefore, at an impact velocity of 86.5 m/s, the digital thoracic target must be calibrated and optimized using the impact response of the physical thoracic target. This ensures consistent impact responses between the physical and digital thoracic targets under non-lethal kinetic energy projectile impacts. It also improves the credibility of the digital thoracic target.

#### 4.2.1 Simulation parameter optimization workflow for the digital thoracic target

Dancerel-Bourlon et al. (2025) found that, during non-lethal kinetic energy projectile impact to the chest, the thoracic skeleton mainly supports chest cage deflection and maintains overall thoracic stability. Therefore, compared with skin, soft tissue, and other components, it can be inferred that the thoracic skeleton can mainly determine the magnitude and rate of chest-wall displacement. The thoracic skeleton consists mainly of the sternum, ribs, and costal cartilage. The skeletal displacement process of the digital thoracic target was examined. After projectile impact, the simulated sternum first received the impact energy and underwent displacement. This then caused compressive deformation of the surrounding costal cartilage. The ribs behind the costal cartilage provided support during cartilage deformation and buffered the overall chest-wall displacement through compression. Therefore, the thoracic skeleton was selected for simulation parameter optimization.

In the CHEST digital thoracic target, the sternum, ribs, and costal cartilage were all assigned material No. 001, *MAT_ELASTIC, which is a linear elastic constitutive model. Its basic parameters include density, Young’s modulus, and Poisson’s ratio. Young’s modulus (E) is an intrinsic property that characterizes the axial tensile and compressive stiffness of a material. It directly determines the deformation capacity and energy dissipation efficiency of the costal cartilage under impact loading. Density (ρ) mainly affects the structural inertial force. Poisson’s ratio (ν) has a limited effect on the mechanical response of nearly incompressible soft-tissue materials. Therefore, Young’s modulus was selected as the optimization parameter. Its value was adjusted to analyze the effect of costal-cartilage stiffness on the chest-wall impact displacement response. To identify the specific adjustment target, a local sensitivity analysis was performed to evaluate the effects of changes in the Young’s moduli of the sternum, ribs, and costal cartilage on chest-wall displacement.

The Young’s modulus parameters of the sternum, ribs, and costal cartilage were denoted as *E*_*1*_, *E*_*2,*_ and *E*_*3*_, respectively. Local sensitivity analysis was performed for the three selected parameters. The sensitivity coefficient *S*_*i*_ of chest-wall displacement was calculated to quantify the influence of each parameter on chest-wall displacement. The calculation formula for *S*_*i*_ is as follows.

$$S_{i}=\frac{\Delta Y_{i}/Y_{0}}{\Delta E_{i}/E_{i0}}$$ (11)

In Equation 11, $\Delta Y_{i}$ denotes the chest-wall displacement when the parameter is set to $E_{i}$ . $Y_{0}$ denotes the baseline chest-wall displacement. $\Delta E_{i}$ denotes the change in Young’s modulus $E_{i}$ , and *E*_*i0*_ denotes the initial value of Young’s modulus.

Based on the baseline model parameters, perturbations of ±10%, ±20%, and ±30% were applied. The sensitivity coefficients of *E*_*1*_, *E*_*2,*_ and *E*_*3*_ were then calculated. The results are listed in Table 10.

**TABLE 10:** Values of the *S*_*i*_ for different disturbance magnitudes.

Amplitude of disturbance	+10%	−10%	+20%	−20%	+30%	−30%
*S*_*1*_	0.125	0.117	0.252	0.229	0.342	0.319
*S*_*2*_	0.083	0.076	0.165	0.152	0.254	0.240
*S*_*3*_	0.352	0.331	0.704	0.663	1.055	0.992

The sensitivity coefficient *S*_*i*_ was examined under different perturbation amplitudes. Within the perturbation range of 10%–30%, *S*_*3*_ was significantly higher than *S*_*1*_ and *S*_*2*_. This indicates that the Young’s modulus of the costal cartilage has a significant effect on chest-wall displacement and is a highly sensitive parameter. The Young’s moduli of the sternum and ribs also affect the displacement, but their effects were weaker than that of the costal cartilage. Therefore, the Young’s modulus of the costal cartilage was selected as the optimization parameter. Its value was adjusted to analyze the effect of costal-cartilage stiffness on the chest-wall impact displacement response.

From a biomechanical perspective, the costal cartilage connects the sternum to the anterior ribs and directly participates in the load transfer from the impact region to the rib cage. Its stiffness, therefore, influences the effective compliance of the anterior chest wall, the magnitude of thoracic compression, and the timing of the displacement response. Consequently, modifying the costal cartilage modulus provides a direct means of adjusting the global anterior thoracic response without changing the anatomical geometry or the overall structure of the model.

Only one parameter was optimized because the experimental comparison used the chest displacement time history as the principal calibration response. Simultaneous optimization of several material parameters using a limited number of response variables could produce parameter correlation and non-unique solutions because different combinations of rib, cartilage, ligament, and soft-tissue properties may generate similar global displacement responses. A single-parameter optimization strategy was, therefore, adopted to reduce parameter coupling, avoid overfitting, and maintain the physical interpretability of the optimized model.

The rib and sternum properties were not included in the optimization because these structures mainly provide the load-bearing framework of the thorax, and their parameters showed lower sensitivity to the global displacement response than the costal cartilage modulus within the investigated ranges. Altering their stiffness could also affect local stress, strain, and fracture predictions, which were not directly measured in the physical experiments.

Ligament parameters were excluded because they primarily influence local joint constraints and relative motion between adjacent skeletal structures. Their effects cannot be uniquely identified using the anterior displacement response alone. Similarly, the thoracic soft tissues comprise several materials with nonlinear and potentially strain rate-dependent behavior. Simultaneously varying these properties would substantially increase the number of optimization variables and introduce strong parameter interactions.

Therefore, the exclusion of these tissues does not imply that they are biomechanically unimportant. Rather, their parameters could not be independently and reliably identified from the available experimental response. The costal cartilage modulus was selected as the most sensitive and mechanically interpretable parameter for the present displacement-based calibration.

To improve the accuracy of material-parameter calibration for the simulated costal cartilage, LS-OPT was used to optimize its Young’s modulus. First, the k file for the CHEST target impacted by the projectile at a velocity of 86.5 m/s was opened in LS-PREPOST. The optimization parameter *E* was entered in the *PARAMETER card, and its initial value was set to 49.0 MPa. Based on the Young’s modulus range of costal cartilage reported in Weber et al. (2021), the optimization range was set to 30–80 MPa. The output options were kept unchanged. During optimization, optimization analysis was selected in Task Settings. The Sequential Response Surface Method (SRSM) was used as the optimization algorithm because it has high computational efficiency and good convergence. In Stage 1, the optimization file path was configured. LS-DYNA was selected as the solver, and the corresponding calculation files were imported. The Global Limit was set to 4. In the Set Up module, Young’s modulus *E* was defined as the only optimization parameter. In Sampling 1, the response surface construction method was set to Polynomial, and the sampling method was set to Linear. The simulated displacement was used as the response variable. In the Optimization module, the physical test curve was defined as the target curve. Because only one optimization variable was used, its target weight was set to 1. For the termination criteria, the maximum allowable error was set to 0.05, and the maximum number of iterations was set to 10. Based on these settings, the Young’s modulus of the simulated costal cartilage was optimized. The complete workflow is shown in Figure 20.

**FIGURE 20:**
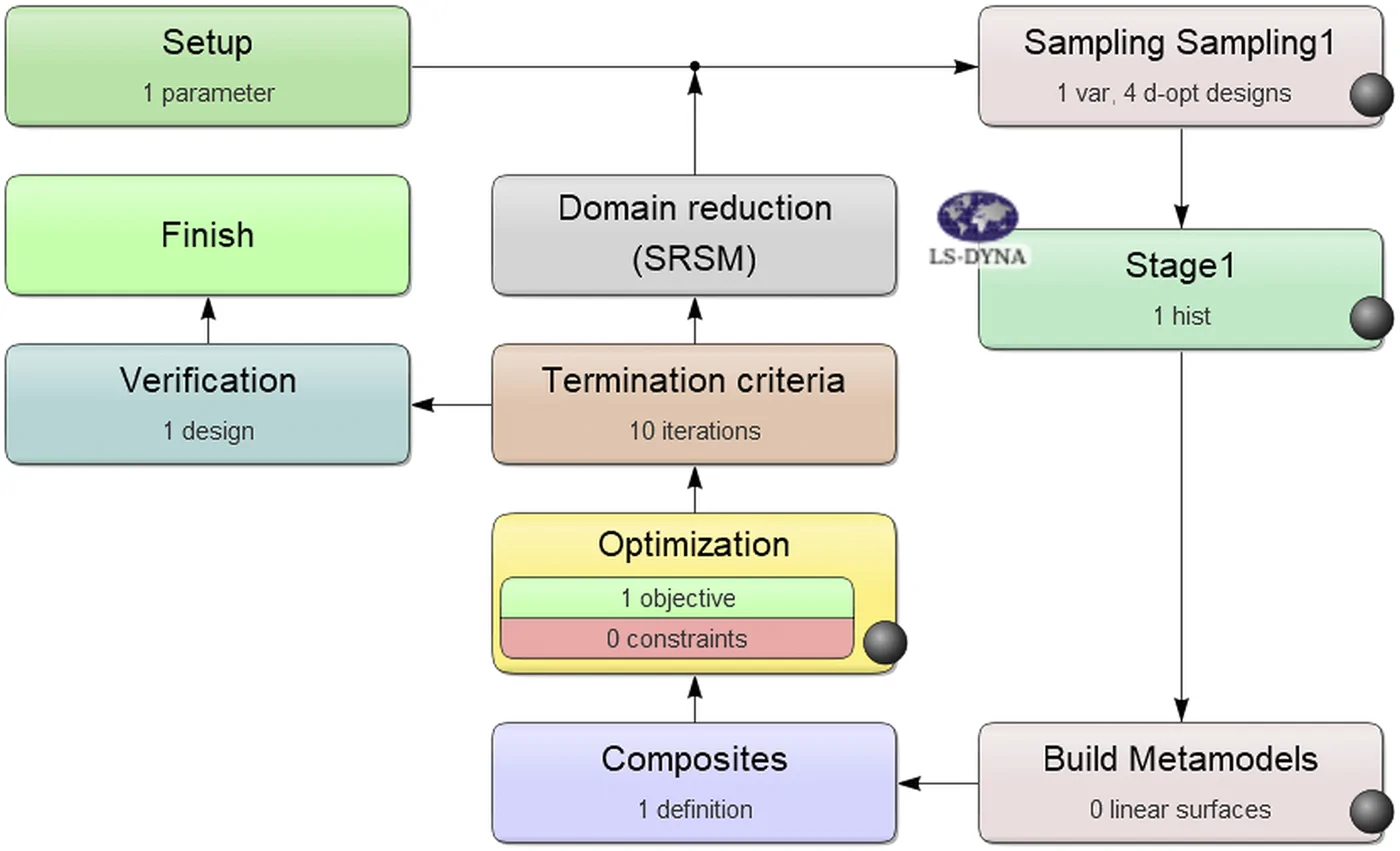
Optimization workflow for the LS-OPT Sequential Response Surface Method (SRSM).

#### 4.2.2 Results of the optimization of simulation parameters for the digital thoracic target

A baseline run was performed for the completed optimization workflow. No abnormality occurred. The workflow was then executed as a normal run. After completion, the optimization process was checked in the History module, and the optimization results were obtained. The results showed that the optimization converged after six iterations. The optimal Young’s modulus *E* was 62.293 MPa, and the optimized displacement–time curve was obtained. The displacement–time curve at an impact velocity of 56 m/s was then recalculated using the optimized parameter. The comparisons before and after optimization are shown in Figure 21.

**FIGURE 21:**
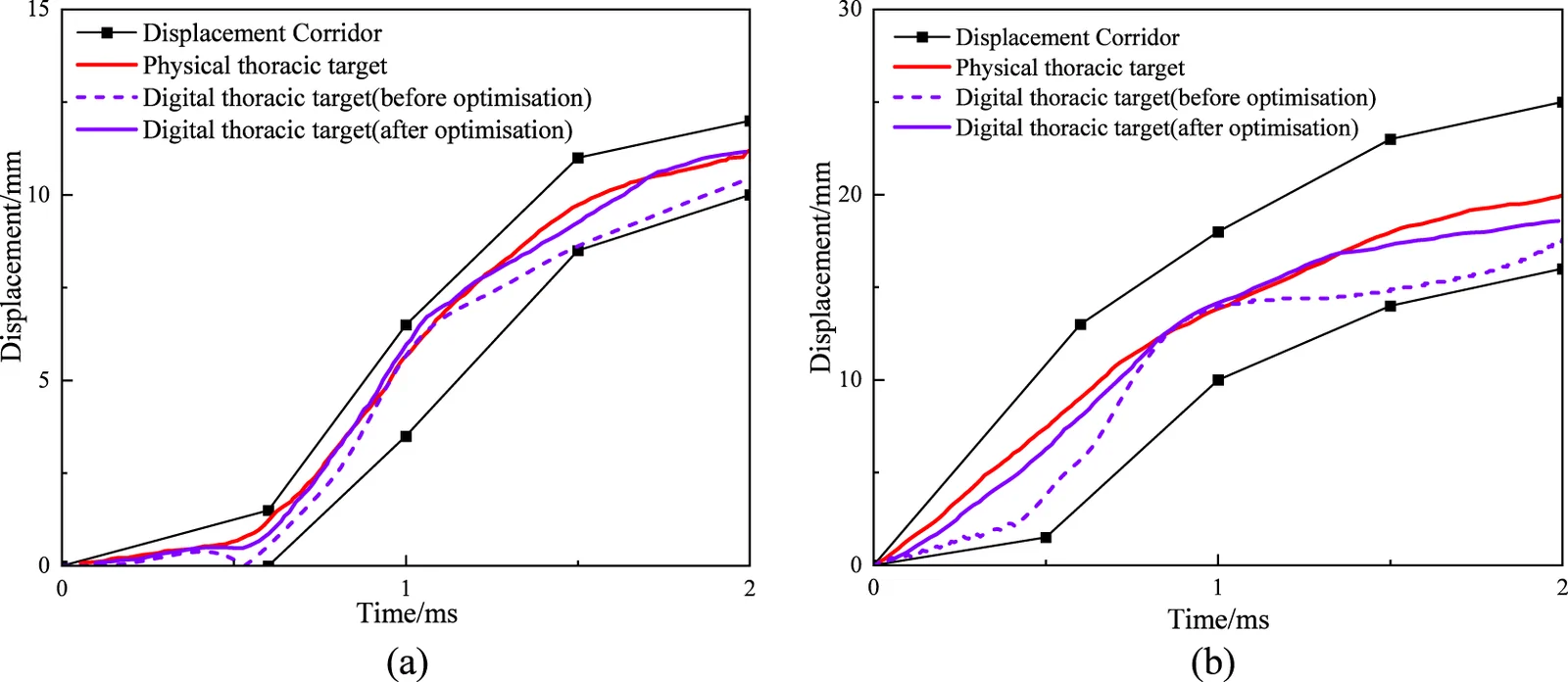
Comparison of displacement–time curves before and after optimization of the digital thoracic target. **(a)** Displacement–time curve (56 m/s). **(b)** Displacement–time curve (86.5 m/s).

Because the displacement–time curve of the optimized digital thoracic target changed, its VC_max_ value was recalculated. The VC_max_ values were 0.307 m/s at projectile impact velocities of 56 m/s and 0.825 m/s at 86.5 m/s. Both values met the standard requirements.

The MAE and NMAE were also calculated to analyze the error between the optimized digital thoracic target and the physical thoracic target. First, the numerical simulation curve and the physical test curve were resampled to the same number of equally spaced data points by linear interpolation. Then, based on Equations 9, 10, the MAE and NMAE between the optimized digital thoracic target simulation and the physical test were calculated. The results are listed in Table 11.

**TABLE 11:** MAE and NMAE values for the displacement of the optimized digital and the physical thoracic target under impact.

Projectile impact velocity (m/s)	MAE (mm)	NMAE (%)
56	0.391	3.4
86.5	0.855	4.9

The MAE and NMAE values of the digital and physical thoracic targets before and after optimization were compared at the two impact velocities. The comparison results are shown in Figure 22.

**FIGURE 22:**
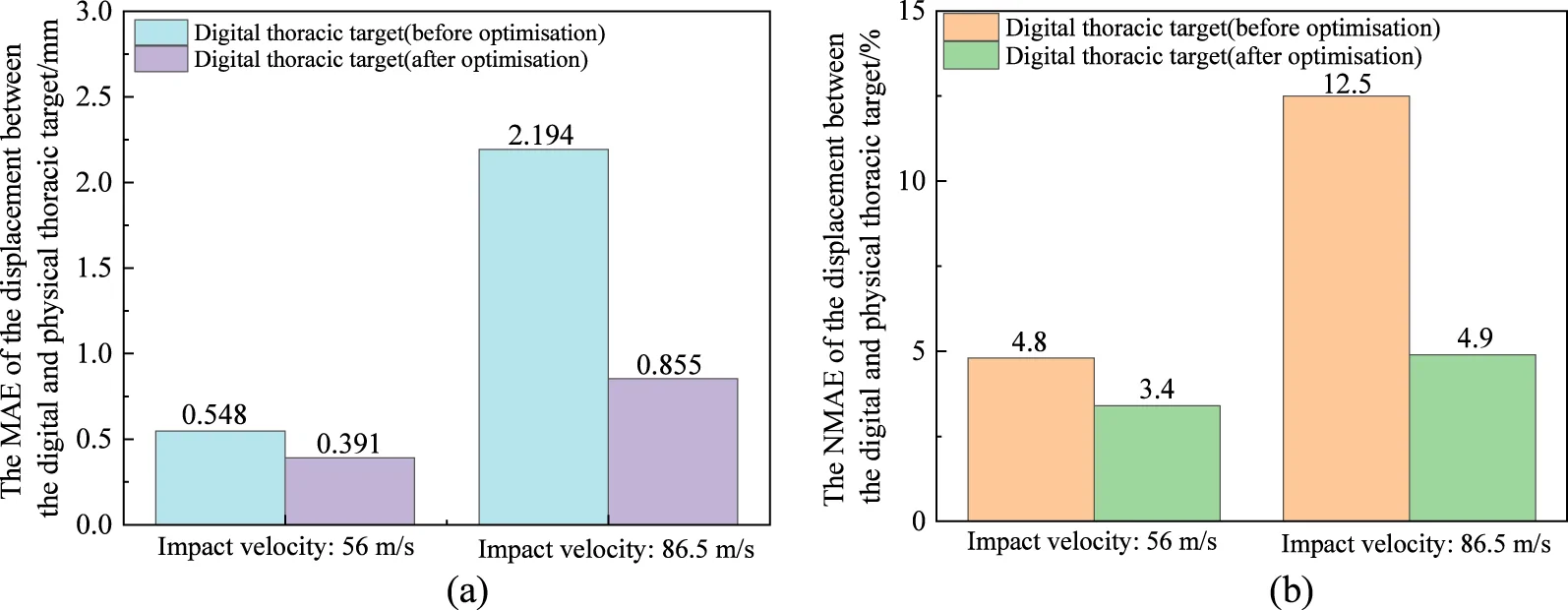
Comparison of MAE and NMAE for impact-induced displacement between digital and physical thoracic targets before and after optimization. **(a)** Comparison of the MAE for impact displacement between digital and physical thoracic targets **(b)** Comparison of the NMAE for impact displacement between digital and physical thoracic targets.

After optimization, the NMAE between the digital and physical thoracic targets decreased to 3.4% at 56 m/s and 4.9% at 86.5 m/s. Both values were below 5%. This indicates that the agreement between the simulation and physical test was greatly improved.

The VC_max_ values of the digital and physical thoracic targets before and after optimization were also compared. The corresponding error values are listed in Table 12.

**TABLE 12:** Comparison of VC_max_ and their errors for the digital and physical thoracic targets before and after optimization.

Thoracic target type	56 m/s	Error relative to the physical thoracic target	86.5 m/s	Error relative to the physical thoracic target
Physical thoracic target	0.291	—	0.841	—
Digital thoracic target (before optimization)	0.319	9.62%	0.785	6.67%
Digital thoracic target (after optimization)	0.307	5.50%	0.825	1.90%

After optimization, the VC_max_ error between the digital and physical thoracic targets decreased from 9.62% to 5.50% under the 56-m/s impact velocity. Under the 86.5-m/s impact velocity, the VC_max_ error decreased from 6.67% to 1.90%. These results show that the optimized digital thoracic target achieved good agreement with the physical thoracic target with respect to both VC_max_ and displacement response.

The improvement in displacement agreement after optimization can be explained by the biomechanical function of the costal cartilage. During anterior blunt impact, the load is transmitted from the sternum and anterior chest wall to the ribs through the costal cartilage. Changing its Young’s modulus alters the effective flexural compliance of the anterior thoracic structure and consequently affects the magnitude and temporal evolution of chest compression.

The optimization should not be interpreted as evidence that the costal cartilage is the only structure controlling the thoracic impact response. The ribs, sternum, ligaments, and soft tissues also contribute to load transmission, deformation, and energy dissipation. However, their parameters had lower sensitivity to the selected global displacement response or could not be uniquely identified from the available experimental measurements. Therefore, the present optimization represents a parsimonious calibration of the most influential identifiable parameter rather than a complete material parameter identification of the thorax.

Direct numerical comparison of the optimized costal cartilage modulus with that reported in previous studies was not possible because the available studies used different thoracic models, loading devices, impact directions, response variables, material formulations, and optimization objectives. Some studies focused on isolated rib properties, some adjusted several skeletal or soft-tissue parameters, and others improved model responses through hierarchical validation or material homogenization rather than inverse optimization.

Nevertheless, the present findings are methodologically consistent with those of previous thoracic model development studies in three respects. First, previous studies have shown that the global thoracic response is sensitive to the mechanical properties of the skeletal and cartilaginous structures. Second, component or parameter sensitivity analysis has commonly been used to identify the structures that have the greatest influence on the selected response before model modification. Third, experimental force, displacement, or deformation responses have been used as targets for evaluating or calibrating finite-element model parameters.

The optimized modulus should not be interpreted as a universally valid material property of human costal cartilage. It represents an effective model calibration parameter associated with the current thoracic geometry, constitutive formulations, boundary conditions, projectile, impact velocities, and displacement-based objective function. Therefore, comparison with previous studies supports the general calibration strategy and the biomechanical role of thoracic material stiffness, whereas the quantitative validity of the optimized value is primarily supported by the improvement in the present physical–numerical response agreement.

The proposed methodology is applicable to improving the response consistency between a physical thoracic target and its corresponding finite-element model under identical and controlled impact conditions. In the present study, the method was applied to the CHEST model and the corresponding physical thoracic target under SIR-X impacts at 56 m/s and 86.5 m/s using the chest displacement history as the optimization response and the costal cartilage Young’s modulus as the selected parameter. Although the optimized model showed improved agreement at both velocities, the calibrated modulus should be regarded as an effective parameter for the current model, projectile, impact conditions, and objective function rather than a universal material property. The current conclusions are, therefore, limited to the investigated frontal impact conditions, and further validation using additional velocities, projectile types, impact locations, and experimental responses is required before broader application.

### 4.3 A study on the safety design of a new type of non-lethal kinetic projectile based on a thoracic target with consistent impact response

After parameter calibration, the digital thoracic target showed small errors in displacement–time curves and VC_max_ values relative to the physical thoracic target under the two impact velocities. This indicates strong agreement between their impact responses. After the response-consistent thoracic target was constructed and validated, it was used in the study of the safety design of a new non-lethal kinetic energy projectile.

Bir and Viano (2004) fitted a logistic injury risk curve between VC_max_ and the probability of moderate-to-severe thoracic injury (AIS 2–3) using PMHS impact tests with non-lethal kinetic energy projectiles, as shown in Figure 23.

**FIGURE 23:**
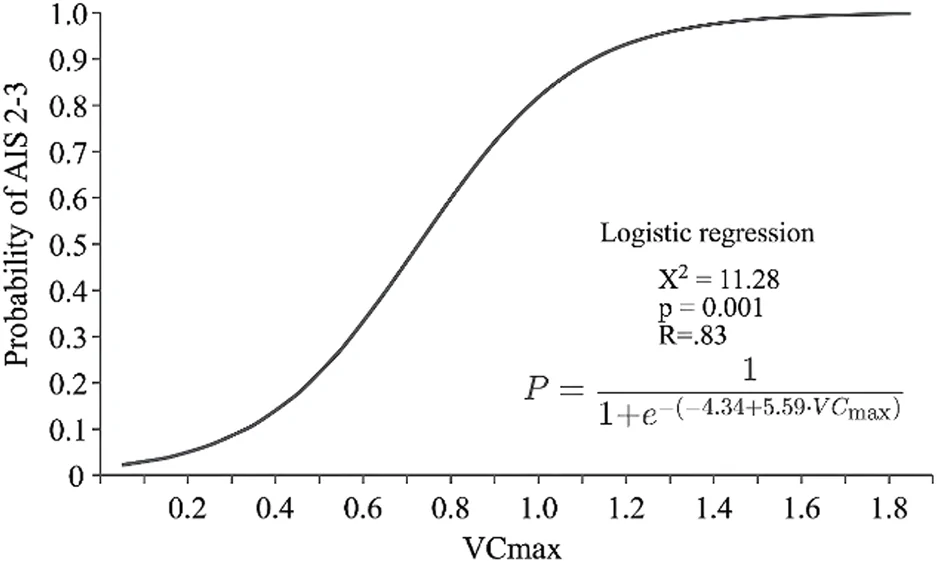
Relationship between VC_max_ and the probability of AIS 2–3 thoracic injury.

Based on the study by Bir and Viano (2004) on projectile impact VC_max_ and thoracic injury risk and the study by Nsiampa (2016) on safe velocities for various non-lethal kinetic energy projectiles, a 5%–50% probability of AIS 2–3 thoracic injury was defined as the safety range. Substituting this range into the logistic injury risk curve showed that the safe VC_max_ range was 0.25–0.8 m/s.

In the safety design of the new non-lethal kinetic energy projectile, the safe VC_max_ range of 0.25–0.8 m/s was used as the criterion. A projectile finite-element model was first constructed from the new projectile geometry. Projectile impact simulations were then performed using the CHEST finite-element model. The safe velocity range [*v*_*1*_ *,v*_*2*_] was obtained when the VC_max_ of the digital thoracic target was 0.25–0.8 m/s. The safe firing-distance range [*x*_*1*_ *,x*_*2*_] was then derived using the relevant equations. If the safe firing distance did not meet the requirements, the geometry, material, and related parameters of the digital projectile were modified. The projectile impact simulation on the digital thoracic target was then repeated. If the safe firing distance met the requirements, the physical projectile was fabricated and tested.

After the geometry and material parameters of the digital projectile were determined, the physical projectile was fabricated using the corresponding material and design parameters. After fabrication, blunt impact tests were conducted by using the physical projectile to impact the 3-rib thoracic model based on the safe velocity range [*v*_*1*_ *,v*_*2*_] determined from the digital projectile. The corresponding VC_max_ values of the physical thoracic target were obtained and compared with the safe range of 0.25–0.8 m/s. If the VC_max_ values from the physical thoracic target were within this range, the design was considered acceptable. If not, the physical projectile material was optimized and adjusted. The safe velocity range of the physical projectile was then obtained as [*v*_*3*_ *,v*_*4*_], and the corresponding firing-distance range was obtained as [*x*_*3*_ *,x*_*4*_].

When both the digital and physical projectiles passed the validation, the new projectile was considered validated. The final safe firing-distance range was determined as [max{*x*_*1*_,*x*_*3*_},min{*x*_*2*_,*x*_*4*_}]. The safety design workflow is shown in Figure 24.

**FIGURE 24:**
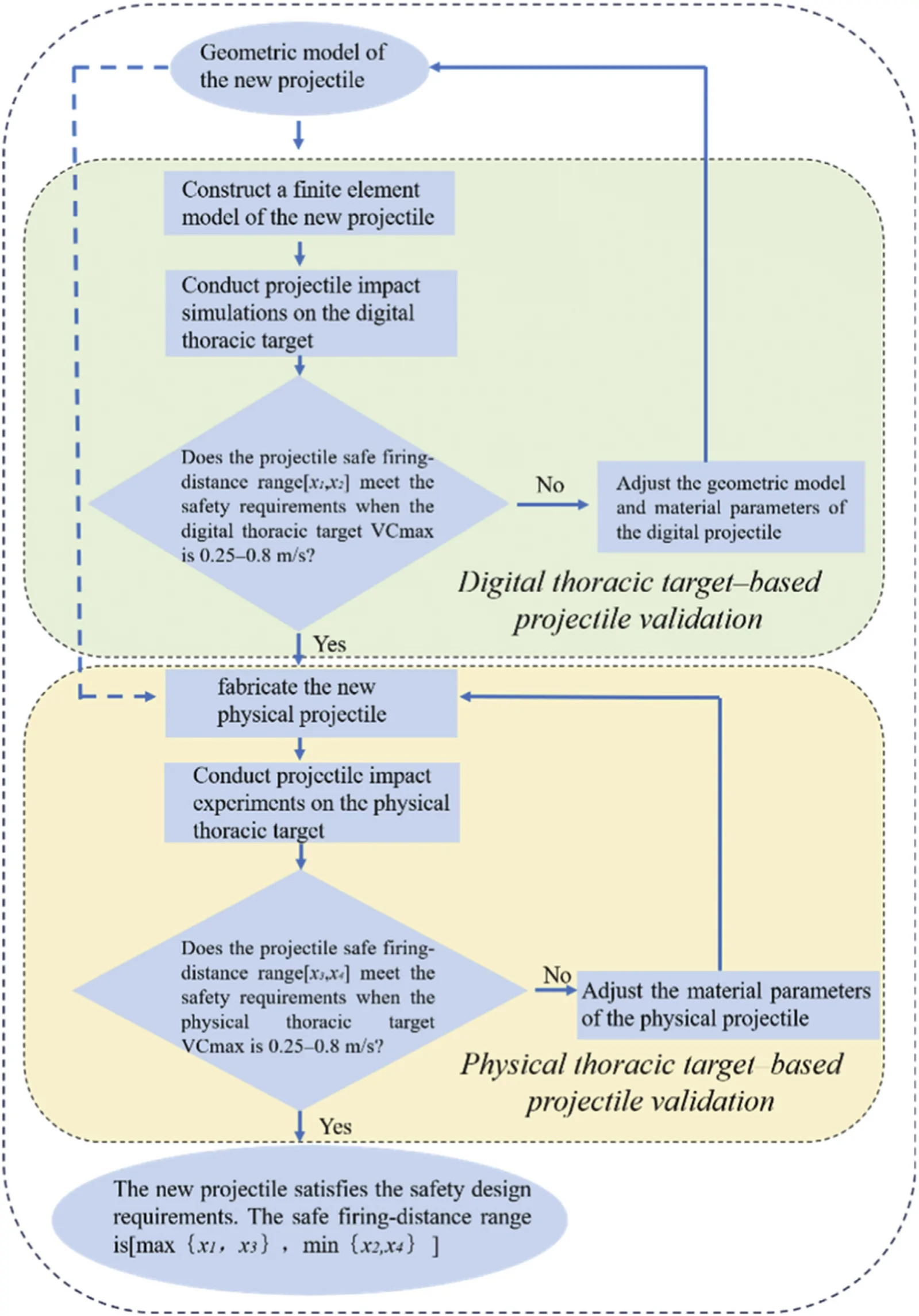
Safety design workflow of the new projectile based on the response-consistent thoracic target.

The safety design workflow for the new projectile based on the response-consistent thoracic target follows a simulation-first and experiment-second procedure. This workflow can effectively reduce uncertainty in the experimental stage and save both time and cost. In the simulation stage, key parameters can be adjusted to optimize the projectile design in advance. This helps ensure that the projectile meets the safety requirements. It also avoids the high cost and time delay caused by repeated adjustments during experiments. Simulation provides multidimensional performance evaluation. It allows different design schemes to be fully assessed before physical testing, thereby improving design accuracy and efficiency. Validation in the physical experimental stage compensates for possible model simplifications and parameter uncertainties in the simulation model. It also supports the scientific validity and feasibility of the digital projectile design. In summary, the safety design workflow for the new projectile based on the response-consistent thoracic target has good economic efficiency, safety, and reliability.

## 5 Applicability and limitations

The effects of the strain rate were not explicitly characterized in the present study. The mechanical properties assigned to the surrogate ribs and several components of the finite-element thoracic model were obtained from nominal values or previously reported data. Consequently, possible changes in stiffness, strength, and failure behavior under high rate loading were not fully represented. Although the physical and finite-element targets were evaluated using their overall displacement and viscous responses under the prescribed impact velocities, agreement at the structural response level does not constitute direct validation of the rate-dependent constitutive behavior of individual materials.

In addition, the optimized Young’s modulus of the costal cartilage should be interpreted as an effective calibration parameter for the current projectile, impact velocities, and model configuration rather than as a universally applicable dynamic material property. Future work will include dynamic material testing of the surrogate rib materials and thoracic tissues, followed by the implementation of strain rate-dependent and damage-capable constitutive models. These improvements will enable further evaluation of local stress, strain, failure, and energy absorption under a wider range of impact conditions.

Regarding the material properties of the organs in the finite-element model, the elastic formulations adopted for the thoracic organs should be regarded as effective approximations for the investigated global responses and loading conditions rather than complete descriptions of organ mechanics. This simplification may limit the accuracy of predicted local tissue deformation, stress, and failure; therefore, these local quantities were not considered independently validated injury indicators. Future studies should incorporate dynamic material characterization and nonlinear, viscoelastic, and strain-rate-dependent constitutive models, together with organ-level experimental measurements, to improve the prediction and validation of local tissue responses under blunt ballistic impact.

The uncertainty analysis in this study was limited to the displacement measurement because the individual time history data from the repeated tests were not available for retrospective statistical analysis. Therefore, the repeatability uncertainty and the complete uncertainty propagation for VC_max_, which depends on numerical differentiation of the displacement signal, could not be recalculated. This limitation should be considered when interpreting the reported response values. Future experiments will retain the complete raw time history data and calibration records to allow a comprehensive uncertainty analysis.

The use of only two impact velocities in the experiments limits the interpretation of the results to the validation conditions prescribed in AEP-99. The present study results cannot establish a continuous relationship between impact velocity and thoracic response or be directly extended to other velocity ranges. Future studies will include additional impact velocities to characterize the velocity-dependent response and further evaluate the applicability of the proposed consistency assessment method.

Contact force and energy transfer were not measured in the physical impact tests. Therefore, these quantities were used only as supplementary numerical indicators for interpreting the impact loading and energy transfer mechanisms. They were not treated as independent experimental validation metrics. Future physical tests incorporating force measurement will be required to validate these numerical responses directly.

Regarding the simulation contact parameter settings, a dedicated sensitivity analysis of the friction coefficient and penalty stiffness parameters was not conducted in the present study. Therefore, although these contact parameters were kept constant throughout all simulations, their potential influence on the predicted local contact response remains a limitation and should be quantitatively evaluated in future work.

Regarding the selection of the regions for parameter adjustment, only the Young’s modulus of the costal cartilage was optimized. Although the Young’s modulus showed the highest sensitivity to the displacement response, other thoracic components also influence the biomechanical response. Because the available physical experiments primarily provided chest displacement data, simultaneous identification of multiple tissue parameters would have been underdetermined and could have produced non-unique parameter combinations. The optimized costal cartilage modulus should, therefore, be interpreted as an effective parameter for the present model, projectile, impact velocities, and calibration objectives rather than as a universally applicable material property. Future studies should incorporate additional experimental responses, such as contact force, local strain, and regional displacement, to support multiparameter calibration and independently evaluate the material properties of the ribs, sternum, ligaments, and soft tissues.

Regarding the comparison with previous studies on parameter optimization of digital thoracic models, direct comparison with previously optimized costal cartilage parameters was limited because no study with the same human thorax model, physical target, SIR-X projectile, AEP-99 impact conditions, and displacement-based optimization objective was identified. Related studies differed in their model geometry, material formulations, loading conditions, and calibration responses. Therefore, the literature comparison in the present study was limited to the optimization methodology, parameter sensitivity, and biomechanical response trends rather than direct comparison of optimized numerical values. In addition, the optimized costal cartilage modulus was evaluated using the same two loading conditions included in the consistency assessment in this study. Independent validation under additional projectile velocities, impact locations, and loading directions is required to determine whether the calibrated parameter can be generalized beyond the current conditions.

## 6 Conclusion

This study established a unified framework for verifying and improving the response consistency between a physical thoracic target and a detailed human thorax finite-element model under standardized blunt ballistic impact. The physical and numerical targets were evaluated under identical AEP-99 loading conditions, and the finite-element model was subsequently calibrated against the experimentally measured chest displacement response.

The main scientific contribution of this study is the integration of standard validation, quantitative physical–numerical response comparison, sensitivity-based parameter selection, and experiment-informed optimization within a single procedure. The findings indicate that independent compliance with the same validation criteria does not necessarily ensure quantitative consistency between physical and numerical targets. The combined interpretation of displacement, contact force, and thoracic internal energy further clarifies the biomechanical sequence of transient loading, chest deformation, energy transfer, and structural recovery.

From an engineering perspective, PA66+GF30 was identified as a suitable candidate rib material for the investigated physical thoracic target, while the proposed optimization procedure improved the response agreement of the CHEST model without changing its anatomical structure. The methodology may support the development and refinement of corresponding physical and numerical thoracic targets for non-lethal projectile assessment, provided that their geometry, boundary conditions, projectile characteristics, and measured responses are appropriately matched.

The conclusions are limited to one physical–numerical target pair, two frontal SIR-X impact conditions, and a displacement-based single-parameter calibration. The optimized costal cartilage modulus should, therefore, be interpreted as an effective parameter for the present model and loading configuration rather than a universal material property. In addition, the numerically obtained contact force, energy response, and local tissue responses were not independently validated through corresponding experimental measurements.

Future studies should include additional impact velocities, projectile types, impact locations, and independent validation cases. Experimental measurements of contact force, regional deformation, and local strain should be introduced to support multiresponse and multiparameter calibration. Dynamic material characterization and strain rate-dependent constitutive models should also be incorporated to improve the prediction of local tissue deformation, stress, and failure under blunt ballistic impact.

## Data Availability

The original contributions presented in the study are included in the article/supplementary material; further inquiries can be directed to the corresponding author.
